# Neutron crystallography and quantum chemical analysis of bilin reductase PcyA mutants reveal substrate and catalytic residue protonation states

**DOI:** 10.1016/j.jbc.2022.102763

**Published:** 2022-12-01

**Authors:** Tatsuya Joutsuka, Ryota Nanasawa, Keisuke Igarashi, Kazuki Horie, Masakazu Sugishima, Yoshinori Hagiwara, Kei Wada, Keiichi Fukuyama, Naomine Yano, Seiji Mori, Andreas Ostermann, Katsuhiro Kusaka, Masaki Unno

**Affiliations:** 1 Graduate School of Science and Engineering, Ibaraki University, Hitachi, Ibaraki, Japan; 2 Frontier Research Center for Applied Atomic Sciences, Ibaraki University, Naka-Tokai, Ibaraki, Japan; 3 Department of Medical Biochemistry, Kurume University School of Medicine, Kurume, Fukuoka, Japan; 4 Department of Biochemistry and Applied Chemistry, National Institute of Technology, Kurume College, Kurume, Fukuoka, Japan; 5 Department of Medical Sciences, University of Miyazaki, Miyazaki, Miyazaki, Japan; 6 Graduate School of Science, Osaka University, Toyonaka, Osaka, Japan; 7 Heinz Maier-Leibnitz Zentrum (MLZ), Technical University Munich, Garching, Germany

**Keywords:** neutron scattering, phycocyanobilin, protonation, absorption spectra, enzyme catalysis, ultraviolet-visible spectroscopy, enzyme structure, hydrogen bond, mutant, 18EtBV, 18^1^,18^2^-dihydrobiliverdin, BV, biliverdin, HOMO, highest occupied molecular orbital, LUMO, lowest unoccupied molecular orbital, PCB, phycocyanobilin, PcyA, phycocyanobilin:ferredoxin oxidoreductase, QM/MM, quantum mechanics/molecular mechanics

## Abstract

PcyA, a ferredoxin-dependent bilin pigment reductase, catalyzes the site-specific reduction of the two vinyl groups of biliverdin (BV), producing phycocyanobilin. Previous neutron crystallography detected both the neutral BV and its protonated form (BVH^+^) in the wildtype (WT) PcyA–BV complex, and a nearby catalytic residue Asp105 was found to have two conformations (protonated and deprotonated). Semiempirical calculations have suggested that the protonation states of BV are reflected in the absorption spectrum of the WT PcyA–BV complex. In the previously determined absorption spectra of the PcyA D105N and I86D mutants, complexed with BV, a peak at 730 nm, observed in the WT, disappeared and increased, respectively. Here, we performed neutron crystallography and quantum chemical analysis of the D105N–BV and I86D–BV complexes to determine the protonation states of BV and the surrounding residues and study the correlation between the absorption spectra and protonation states around BV. Neutron structures elucidated that BV in the D105N mutant is in a neutral state, whereas that in the I86D mutant is dominantly in a protonated state. Glu76 and His88 showed different hydrogen bonding with surrounding residues compared with WT PcyA, further explaining why D105N and I86D have much lower activities for phycocyanobilin synthesis than the WT PcyA. Our quantum mechanics/molecular mechanics calculations of the absorption spectra showed that the spectral change in D105N arises from Glu76 deprotonation, consistent with the neutron structure. Collectively, our findings reveal more mechanistic details of bilin pigment biosynthesis.

Bilin pigments, which are open-chain tetrapyrroles biosynthesized from heme, are widely utilized in photosynthetic organisms (1). The numerous bilin pigments with different absorption bands contained within the phycobilisome enable efficient photosynthesis by utilizing light over a wide range of wavelengths in red algae and cyanobacteria (2, 3). Phytochromes in plants, cyanobacteriochromes in cyanobacteria, and bacteriophytochromes in other bacteria also contain bilin pigments with variable light absorption characteristics, controlling several light-dependent biological events, including phototaxis, germination, shade avoidance, and flowering (4, 5, 6, 7, 8). The colors or the absorbed light wavelength of pigments bound to proteins are controlled by the chemical characteristics and conformations of the pigments, as well as environmental factors such as pH and adjacent residues (9, 10). Ferredoxin-dependent bilin reductases are involved in the biosynthesis of bilin pigments (11, 12, 13). For instance, phycocyanobilin (PCB), which is a bilin pigment contained in phycobilisome and cyanobacteriochrome, is biosynthesized by a phycocyanobilin:ferredoxin oxidoreductase (PcyA, EC 1.3.7.5) that uses biliverdin (BV) as the substrate. PcyA catalyzes a two-step reduction reaction: in the first step, the D-ring vinyl group of BV is reduced using two electrons and two protons to produce the reaction intermediate, 18^1^, 18^2^-dihydrobiliverdin (18EtBV), and in the second step, the A-ring of 18EtBV is reduced using two electrons and two protons to synthesize PCB (Fig. 1*A*) (14, 15). The reaction catalyzed by PcyA is strictly controlled to aid reduction of the appropriate sites in the correct order.

**Figure 1:**
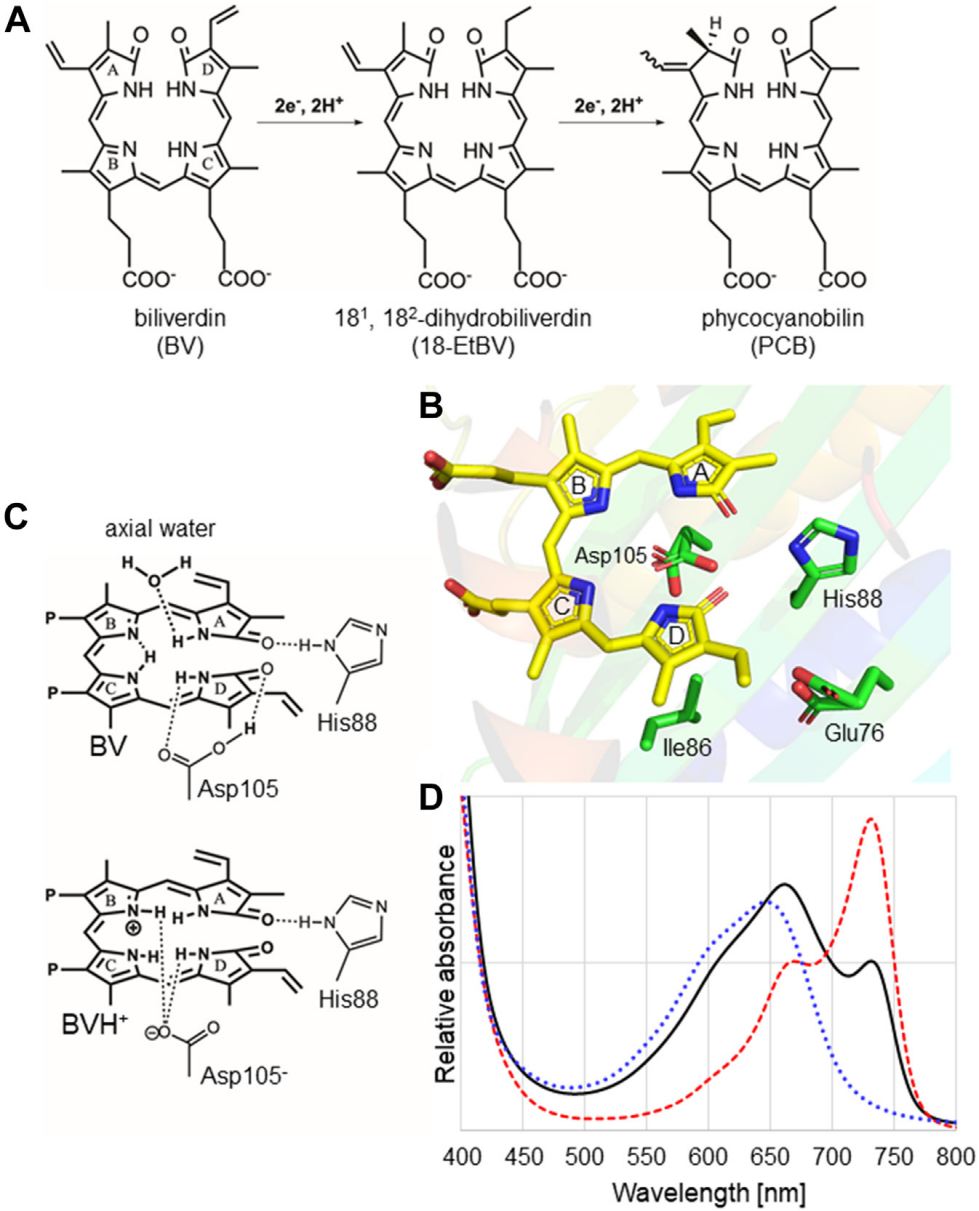
**Unique features of PcyA.** (*A*) The reaction catalyzed by PcyA. (*B*) Structure and locations of important amino acid residues around the substrate BV in the PcyA-BV complex. (*C*) Mixed protonation states in the wildtype PcyA–BV complex. (*D*) Absorption spectra (visible light region) of the wildtype and two PcyA mutants with their substrate BV complexes. The absorption spectra (visible light region) of the WT–BV complex, I86D–BV complex, and D105N–BV complex are depicted with a *solid black line*, *red dashed line*, and *blue dotted line*, respectively.

Mutational studies, spectroscopies, X-ray crystallography, and neutron crystallography have been used to study the unique reaction mechanism of ferredoxin-dependent bilin reductases (11, 16, 17, 18, 19, 20, 21, 22). In the case of PcyA, three residues, namely, glutamate, histidine, and aspartate (Glu76, His88, and Asp105 in *Synechocystis* PcyA), present near the D- and A-rings of BV, are essential for the PcyA reaction (Fig. 1*B*) (18, 23, 24). Among them, Asp105 has two conformations with different protonation states, namely, protonated (-COOH) and deprotonated (-COO^−^) (Fig. 1*C*), which contributes to the transfer of protons to or from BV (25). Furthermore, approximately half of the BV bound to PcyA is positively charged, with all four pyrrole N atoms in protonated states. The protonated state of BV (BVH^+^) complements the deprotonation of the adjacent Asp105, and the positively charged BVH^+^ is considered to facilitate electron acceptance. Finally, one-electron reduction generates a neutral BVH radical, which promotes the PcyA reaction ahead (25).

The D105N mutant, in which aspartate at position 105 is substituted with asparagine, was reported to be only approximately 10% as active as wildtype (WT) PcyA (23). Spectroscopic analysis also revealed that D105N receives an electron to produce BV radicals. However, because of the mutation, enzymatic catalysis does not proceed, as the proton transfer from the carboxy side chain of Asp105 to the substrate is no longer possible (23). Glu76 is also considered the proton donor in the first reaction step (24), showing two conformations in the X-ray structure at cryogenic temperature (hereafter referred to as the “cryo” X-ray structure) in the WT PcyA (Fig. 1*B*) (18). However, the protonation states of the two conformers of Glu76 have not been determined (25). His88 was revealed to be protonated and tilted toward the A-ring lactam O1 atom of BV in the WT PcyA and is considered to transfer a proton to the O atom of the lactam group in the A-ring of BV, thus generating a lactim state. The proton is then suggested to be transferred from the lactim O1 to the lactam O19 of the D-ring during the first reaction step (25, 26).

We recently found that Ile86, located near Glu76 and Asp105 in the three-dimensional (3D) structure (Fig. 1*B*), is important for the PcyA catalytic reaction (27). Although Ile86 is not a catalytic residue, its mutation to aspartic acid (I86D) disrupts the proton relay to BV. Thus, the reaction stops, and in I86D, similar to D105N, (an) electron(s) is/(are) donated to BV (27). Therefore, even though BV radicals are generated, PCB cannot be produced in I86D or D105N. Notably, these amino acid residues (Glu76, His88, Asp105, and Ile86) are highly conserved in the PcyA family (Fig. S1).

During research to elucidate the reaction mechanism of PcyA, we also found that the I86D–BV complex exhibits a unique absorption spectrum with a sharp, high absorption peak around 730 nm (27). This peak is present as a shoulder peak in the WT PcyA–BV complex (hereafter referred to as the “WT–BV complex”), disappearing in the D105N–BV complex (Fig. 1*D*) (27). Based on computational analysis, the differences in the absorption spectra of the PcyA–BV complexes were considered to reflect differences in the protonation states of BV (28). Our results of neutron crystallographic analysis of the WT–BV complex supported the hypothesis that the protonation states of BV correlate with the absorption spectra (25, 28). Although the high-resolution cryo X-ray structures of the D105N–BV and I86D–BV complexes have already been reported and have shown some differences from those of the WT–BV complex, these structures cannot reveal the protonation states in the molecules (27, 29). For instance, in our recent study of the X-ray crystallographic analysis of I86D–BV, we were unable to clearly determine the sites of hydrogen atoms, despite an extremely high resolution of 1.05 Å; we had no choice but to infer the protonation states of the active site from interatomic distances of carboxy groups (COOH or COO^−^) of acidic residues and C=O (or C-OH) groups in the A- and D-rings of BV (27). To confirm that the absorption spectra of the PcyA–BV complex correlate with the protonation states of BV, it is crucial to experimentally determine the protonation states of the two mutants, especially those of BV in those structures, as the current cryo X-ray structures are not sufficient to observe hydrogen atoms.

X-ray crystallography has been employed as a powerful technique to elucidate the reaction mechanism of enzymes in structural biology. In recent years, cryo-EM has become a promising method to reveal the 3D structure of proteins, including enzymes; however, it is not a realistic option for relatively small protein molecules, such as PcyA. In X-ray crystallography, X-rays interact with electrons in the crystal and are scattered; thus, the visibility of atoms depends on their atomic numbers. Since each hydrogen atom contains only one electron, the signals obtained from hydrogen atoms are very weak, making the identification of hydrogen atoms in proteins very difficult. However, hydrogen atoms are often involved in enzymatic reactions, especially those involving redox reactions, *e.g.*, the reaction of PcyA. Experimentally determining the protonation states of amino acids, especially in the active site, and those of the substrate molecules is important for understanding such enzymatic redox reactions. A powerful technique to address this dilemma is neutron crystallography. Neutrons interact with the atomic nuclei, and their scattered intensities are independent of atomic numbers. The hydrogen nucleus shows a high incoherent scattering cross section, leading to an increased background for measurement. Hydrogen atoms in polarized bonds can be replaced by deuterium using crystallization solutions on a D_2_O basis. The deuterium atom has a neutron scattering length comparable with that of a carbon atom, making neutron crystallography the best method for identifying the location of the hydrogen atoms in proteins to date. The combination of X-rays and neutrons can distinguish hydrogen (deuterium) atoms from carbon, oxygen (O), nitrogen (N), and other atoms in proteins and uniquely determine their protonation states, orientations of water molecules, and the donors and acceptors in hydrogen bonds. Therefore, the information obtained from neutron crystallography can be quite helpful in elucidating the reaction mechanism of redox enzymes, such as PcyA. However, neutron crystallography is labor intensive, as it requires large crystals.

In this study, we elucidated the neutron crystallographic structures of the PcyA D105N–BV and I86D–BV complexes to determine the positions of the hydrogen atoms in these structures. In addition, computational analysis using the quantum mechanics/molecular mechanics (QM/MM) method was performed, in conjunction with neutron structural analyses. The protonation states of BV, the surrounding residues, and water molecules in these mutants were also determined. The correlation between the absorption spectra and the protonation state of BV was proven by structural comparison with the WT–BV complex. Furthermore, we discuss the structural factors that affect the reaction of PcyA and why the mutations prevent proton transfer.

## Results and discussions

### Neutron structures of D105N–BV and I86D–BV at room temperature

The neutron structures of PcyA D105N–BV and I86D–BV at room temperature were refined to 2.10 Å and 2.00 Å resolutions, respectively. The *R*-factor and free *R*-factor for the D105N–BV structures were 16.51% and 18.00%, respectively, while those for the I86D–BV structures were 17.25% and 20.82%, respectively. For the D105N–BV and I86D–BV neutron structures, 2045/851 and 2045/750 hydrogen/deuterium atoms, respectively, were included in the structure models (Table 1).

**Table 1:** Statistics for structure refinements

	D105N-BV		I86D-BV	
PDB ID	7YKB		7YK9	
Methods	Neutron	X-ray	Neutron	X-ray
Resolutions (Å)	21.12–2.10	34.58–1.38	29.5–2.00	36.88–1.90
*R*_work_	0.1651	0.1501	0.1725	0.1373
*R*_free_	0.1800	0.1721	0.2082	0.1707
No. of molecule in an asymmetric unit	1		1	
No. of water molecules	219		154	
RMS bond distances (Å)	0.0162		0.0170	
RMS bond angles (°)	1.469		1.510	
No. of D atoms	851		750	
No. of H atoms	2045		2045	
Ramachandran plot, most favored (%)	98.74		97.93	
Ramachandran plot, outlier (%)	0.42		0.00	

No, number.

When compared with the neutron structure of WT–BV at room temperature (PDB ID: 4QCD), the root mean square deviation (RMSD) for Cα atoms (residue numbers 11–240) for the neutron structure of D105N–BV was 0.081 Å and that for I86D–BV was 0.107 Å. At the main chain level, the structures were almost identical to WT–BV, with little effect of single residue mutation. Comparing the room-temperature neutron structure of D105N–BV with the cryo X-ray structure (PDB ID: 3F0L), the RMSD between the Cα atoms of both the structures was 0.203 Å. When comparing the room-temperature neutron structure of I86D–BV with the cryo X-ray structure (PDB ID: 5B4H), the RMSD was 0.245 Å. Owing to these differences between the neutron structures at room temperature and the X-ray structures at cryogenic temperature and the difference in their resolutions, the numbers of water molecules were 219 (room temperature neutron at 2.1 Å resolution) and 298 (cryogenic temperature X-ray at 1.3 Å resolution, PDB ID: 3F0L) for the D105N–BV structure and 154 (room temperature neutron at 2.0 Å resolution) and 378 (cryogenic temperature X-ray at 1.1 Å resolution, PDB ID: 5B4H) for the I86D–BV structure. The differences in the important water molecules are discussed later in the study.

### Protonation states of the substrate BV in D105N–BV and I86D–BV

**Figure 2:**
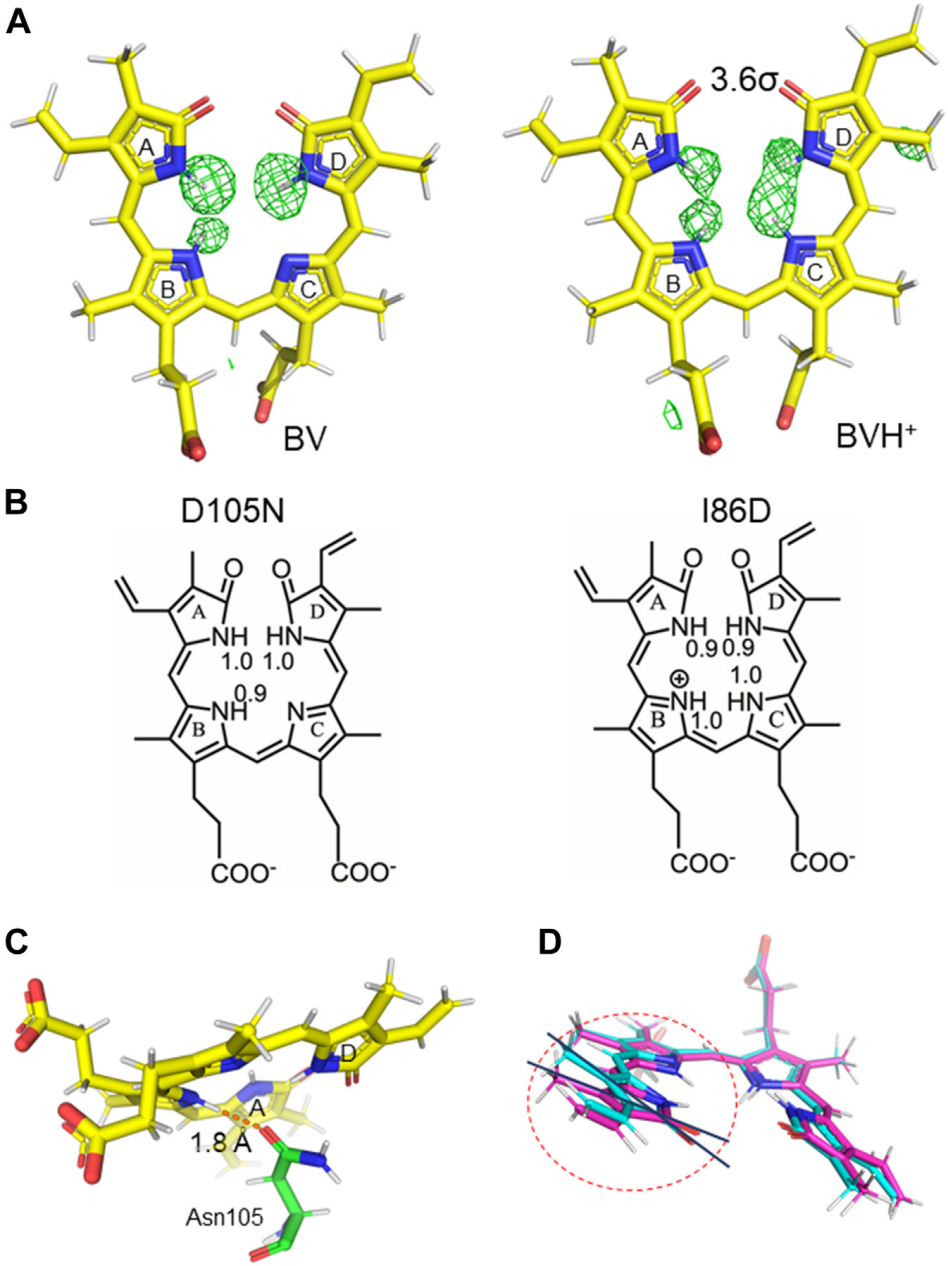
**Structure and protonation states of BV.** (*A*) The *F*_o_–*F*_c_ neutron scattering length density omit maps (*green* meshes) around the substrate BVs, at 3.6σ contour levels, and the structural models. The *left* and *right* panels show BVs in the D105N–BV complex and I86D–BV complex, respectively. In the map calculations, the deuterium and hydrogen atoms of the BV pyrrole *rings* were omitted from the final models. (*B*) Refined occupancies of hydrogen atoms (deuterium atoms) bound to the four BV pyrrole N atoms. It should be noted that all the deuterium atoms on the BV pyrrole N atoms were originally hydrogen atoms. The *left* and *right* panels show BVs in the D105N–BV complex and I86D–BV complex, respectively. (*C*) In D105N–BV, the hydrogen (deuterium) atom of the BV B-ring pyrrole is pulled by a strong hydrogen bond with Asn105. (*D*) Slight differences in the orientations of the D-*rings* of BVs in D105N–BV (*cyan*) and I86D–BV (*magenta*).

The sites of focus in this study were the protonation states of the substrate BV in the structures of D105N–BV and I86D–BV. The *F*_o_–*F*_c_ neutron scattering length density maps, calculated without the hydrogen atoms bound to the pyrrole N atoms in BV in the structures of D105N–BV and I86D–BV, showed a clear difference between the two structures (Fig. 2*A*).

In the structure of D105N–BV, *F*_o_–*F*_c_ neutron scattering length density map features were observed at three pyrrole N atoms, other than the N atom of the C-ring of BV (Fig. 2*A*). This clearly indicated that, in the D105N–BV complex, BV was present only in the neutral state. This is different from the neutron structure of WT–BV, in which BV shows two protonation states, namely, neutral BV and protonated state BVH^+^, each in a different conformation within one molecule. In D105N–BV, the occupancies of deuterium atoms bound to the three pyrrole N atoms other than the C-ring of BV, namely, those on the A-, B-, and D-rings of BV, were determined to be 1.0, 0.9, and 1.0, respectively (Fig. 2*B*). The reason for the slightly lower occupancy of the deuterium atoms bound to the B-ring, when compared with the deuterium atoms in the A- and D-rings, is that the deuterium atom in the B-ring was slightly out of the pyrrole ring plane and oriented toward the O atom of Asn105, possibly forming a strong hydrogen bond (Fig. 2*C*). It is possible that this strong hydrogen bond prevented the hydrogen/deuterium exchange in the B-ring of BV in the crystal and that the hydrogen atoms were not completely replaced by deuterium atoms, thereby leaving a few behind, which would explain why the neutron scattering length density maps corresponding to deuterium (positive) and hydrogen (negative) appeared to cancel each other out, as well as why the occupancy appeared to be low.

On the other hand, the *F*_o_–*F*_c_ neutron scattering length density maps, where the deuterium atoms on the four pyrrole N atoms were omitted around the BV of I86D–BV for the map calculation, showed a similar trend to that of WT–BV (Fig. 2*A*). In other words, residual neutron scattering densities were observed near the N atoms in all the four pyrrole rings of BV. However, careful refinement of the occupancies of these deuterium atoms revealed that the occupancy of the deuterium atom bound to the B-ring pyrrole N and the occupancy of the deuterium atom bound to the C-ring pyrrole N was 1.0 + 1.0 = 2.0 (Fig. 2*B*). Unlike the WT–BV complex, in which BVH^+^ and BV were mixed, in the I86D–BV complex, BVH^+^ was dominantly present, and the presence of neutral BV was insignificant. The presence or absence of this single hydrogen (deuterium) atom caused a slight difference in the conformation of BV (Fig. 2*D*), potentially due to the additional interaction between the hydrogen (deuterium) atoms of the C- and D-rings.

### Interactions between BV and the surrounding residues and the nearby water molecules in the D105N–BV complex

**Figure 3:**
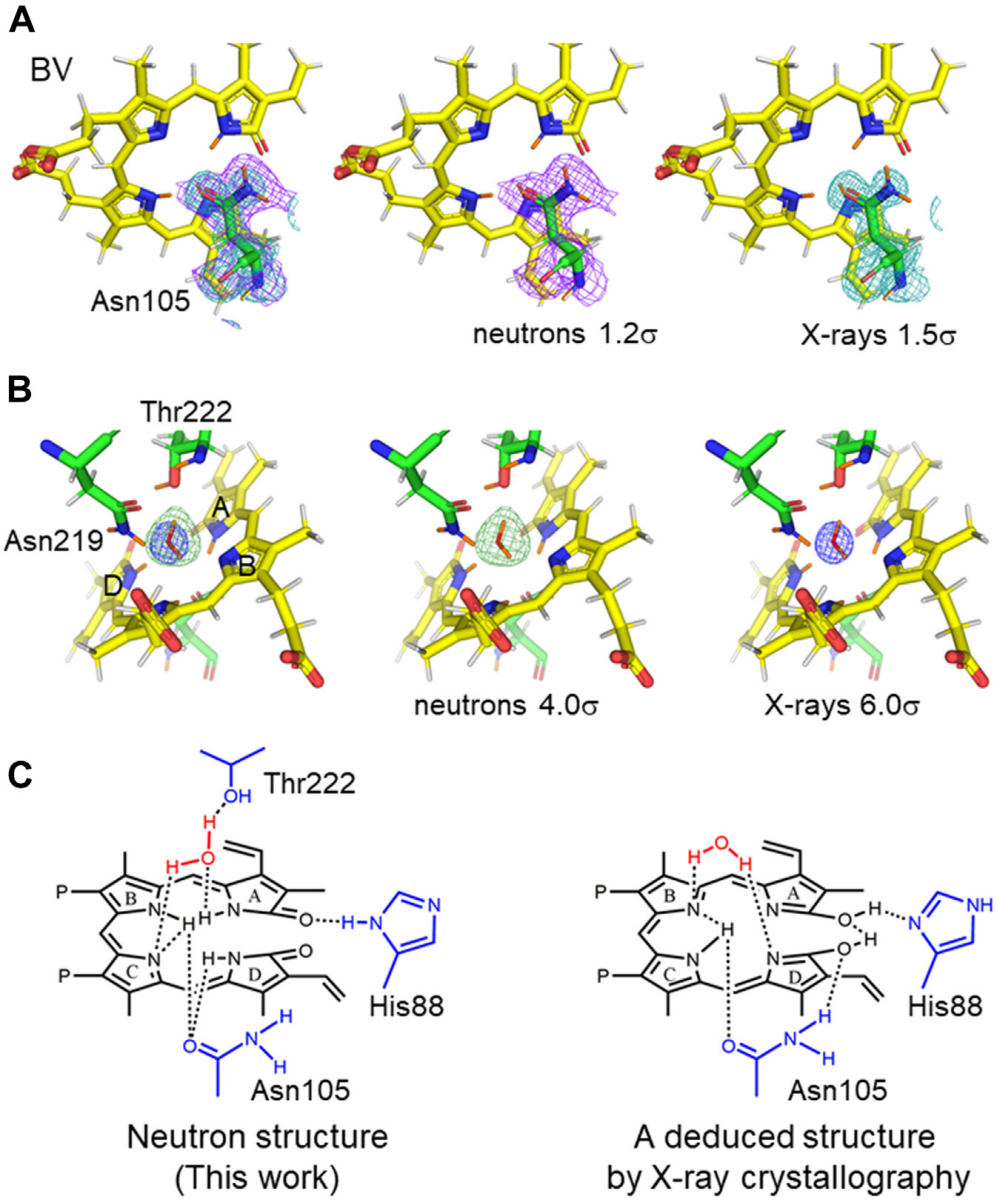
**Structure and protonation states around BV in the D105N–BV complex.** (*A*) The 2*F*_o_–*F*_c_ neutron scattering density (*purple* mesh, 1.2σ contour level) and electron density (*cyan* mesh, 1.5σ contour level) maps for Asn105 in the room temperature D105N–BV, and the structure model determined in the present study. From *left* to *right*: superimposition of both maps, neutron scattering length density map, and electron density map. The structural models are identical. Carbon atoms are colored *yellow* and *green* for BV and Asn105, respectively. The N and O atoms are colored *blue* and *red*, respectively. Hydrogen and deuterium atoms are colored *white* and *orange*, respectively. (*B*) Existence of the axial water molecule in D105N–BV. The *F*_o_–*F*_c_ neutron scattering length density (*green* mesh, 4.0σ contour level) and electron density (*blue* mesh, 6.0σ contour level) omit maps and the structural models. The calculation was performed by omitting the axial water molecule from the final model. The order of displays and color representations are the same as in (*A*). (*C*) Chemical structures of hydrogen-bonding interactions between BV, the axial water molecule, and the surrounding residues. The *left* and *right* panels show the structures in this study and one of the speculations based on the previous cryo X-ray structure (29), respectively.

In the WT–BV complex, Asp105 adopts two conformations: protonated and deprotonated. The two protonation states of Asp105 (neutral Asp105 and Asp105^−^) were considered to correspond to neutral BV and BVH^+^, respectively (18, 25). However, in the D105N–BV complex, both the electron density map and neutron scattering length density map clearly showed that Asn105 was present in a single conformation, with the O atom of the side chain oriented toward BV (Fig. 3*A*). This result differs from that of the cryo X-ray structure of the D105N–BV complex, in which Asn105 was reported to be in a double conformation, although the conformational difference was small (29). The reason for this difference is unclear, and we could not conclude that there are two distinct conformations of Asn105 after X-ray structural analysis at cryogenic temperatures. This could be due to the suppression of X-ray irradiation in this analysis, carried out to help minimize X-ray-induced photoreduction and/or side reactions. Alternatively, the previous cryo X-ray structure showed a spreading electron density, which could have originated from noise, leading to the interpretation of Asn105 as a double conformation (Fig. S2). Alternatively, it could be due to subtle differences in the crystallization conditions and other experimental conditions, such as different cooling rates in the cooling process of the crystals. The neutron crystallographic analysis of D105N–BV at room temperature showed that Asn105 is clearly in a single conformation, consistent with the existence of only neutral-state BV. Furthermore, the significance of this neutron crystallographic analysis was that the O atom and NH_2_ in the side chain of Asn105 could be clearly distinguished, and its conformation was unambiguously determined (Fig. 3*A*).

A water molecule near BV (“axial water”), found in the cryo X-ray structure of the D105N–BV complex, was also present in the room temperature neutron structure; furthermore, the orientation of the axial water molecule could be determined from the neutron scattering length density map (Fig. 3*B*). One hydrogen (deuterium) atom of the axial water molecule was oriented toward the O atom of Thr222, and the other hydrogen (deuterium) atom was oriented toward the C-ring pyrrole N atom. The O atom of this axial water molecule acts as a proton acceptor in the hydrogen bond with the NH_2_ of Asn219. In neutral BV, hydrogen (deuterium) atoms are bonded to the A-, B-, and D-ring pyrrole N atoms and no hydrogen (deuterium) atoms are bonded to the C-ring pyrrole N atom; therefore, space is present only around the BV C-ring pyrrole N atom, and the axial water molecule is considered to adopt a conformation in which one hydrogen atom is directed toward the C-ring pyrrole N atom (Fig. 3*B*). The O atom of the axial water is hydrogen bonded to the pyrrole N atom of the BV A-ring. The previous cryo X-ray structure of D105N–BV could not identify the hydrogen atoms. Although the existence of this axial water molecule is known, the orientation of the water molecule cannot be determined from the X-ray structure. The orientation of the axial water molecule revealed in this study differs from both the orientations inferred from the previous X-ray structure of D105N–BV, in which two hydrogen atoms of the water molecule were not visible but were speculated to be bonded to two pyrrole rings of the BV (Fig. 3*C*) (29).

His88Nδ in D105N–BV was protonated, as seen in WT–BV; however, His88Nε was interpreted as not protonated and His88 was neutral in D105N–BV, which is discussed later in the study (Fig. S3). His88 formed a hydrogen bond with the lactam O atom of the BV A-ring (Fig. 3*C*). The lactam O atom of the A-ring forms a hydrogen bond with another water molecule.

By visualizing the hydrogen atoms that are not visible in X-ray crystallography, we identified the modes of hydrogen bonding interactions between the substrate BV and surrounding residues. Although D105N is regarded an inactive mutant, it actually retains approximately 10% activity (23). This may be attributed to the newly obtained neutron structure at the hydrogen atom level in this study. Our neutron crystallographic analysis of the WT PcyA suggested that Asp105 simultaneously donates and accepts protons to and from BV, respectively (25). Asp105 is considered involved in the reduction of the BV D-ring and the A-ring. The present neutron crystallographic analysis of D105N–BV showed that the axial water molecule could play the role of Asp105 in WT; although less efficiently than Asp105, hydrogen bonds between axial water and the C-ring pyrrole N atom of BV and between axial water and Thr222 could transfer the proton. Previous X-ray crystallographic analysis of D105N–BV, which considered two hydrogen atoms of the water molecule to form a cross-link between the B- and D-pyrrole rings (29), could not explain the 10% activity. Furthermore, the chemical structure inferred from the X-ray structure and spectroscopic results suggested that BV was in the bislactim state (29), whereas our neutron structures of the two mutants (this work) and WT of PcyA showed that BVs were present in the lactam states (25). These results showed that differences in hydrogen bonding modes lead to differences in possible reaction mechanisms. In D105N–BV, axial water was present at almost the same position as that in WT–BV; however, its orientation was different; thus, its role might also be different from that in WT. The neutron structure of the D105N mutant also supported the presence of BV in the lactam state prior to the reaction in PcyA and contributes to why the activity was weak but not zero in the D105N mutant. Thus, visualization of hydrogen atoms is very valuable to explain the efficiency and mechanism of enzymatic reactions.

### Interactions between BV and the surrounding residues and nearby water molecule in the I86D–BV complex

The axial water molecule near the BV found in D105N–BV was not observed in the neutron or high-resolution cryo X-ray structures of the I86D–BV complex (27). One potential reason why the axial water molecule was not observed is that, in BVH^+^ in I86D–BV, a hydrogen (deuterium) atom is bonded to the C-ring of BV, making it difficult for the water molecule to enter due to steric hindrance. The neutron crystallographic structure of WT–BV showed that the corresponding axial water molecule was present at an occupancy of 0.5 (25). The results showing the presence of axial water in the neutron structures of D105N–BV, and not in I86D–BV, validate our hypothesis that the axial water is present only when neutral BV (in which no hydrogen atom is bonded to the C-ring pyrrole N atom creating a vacant space) is present, as proposed in the WT–BV neutron structure analysis.

**Figure 4:**
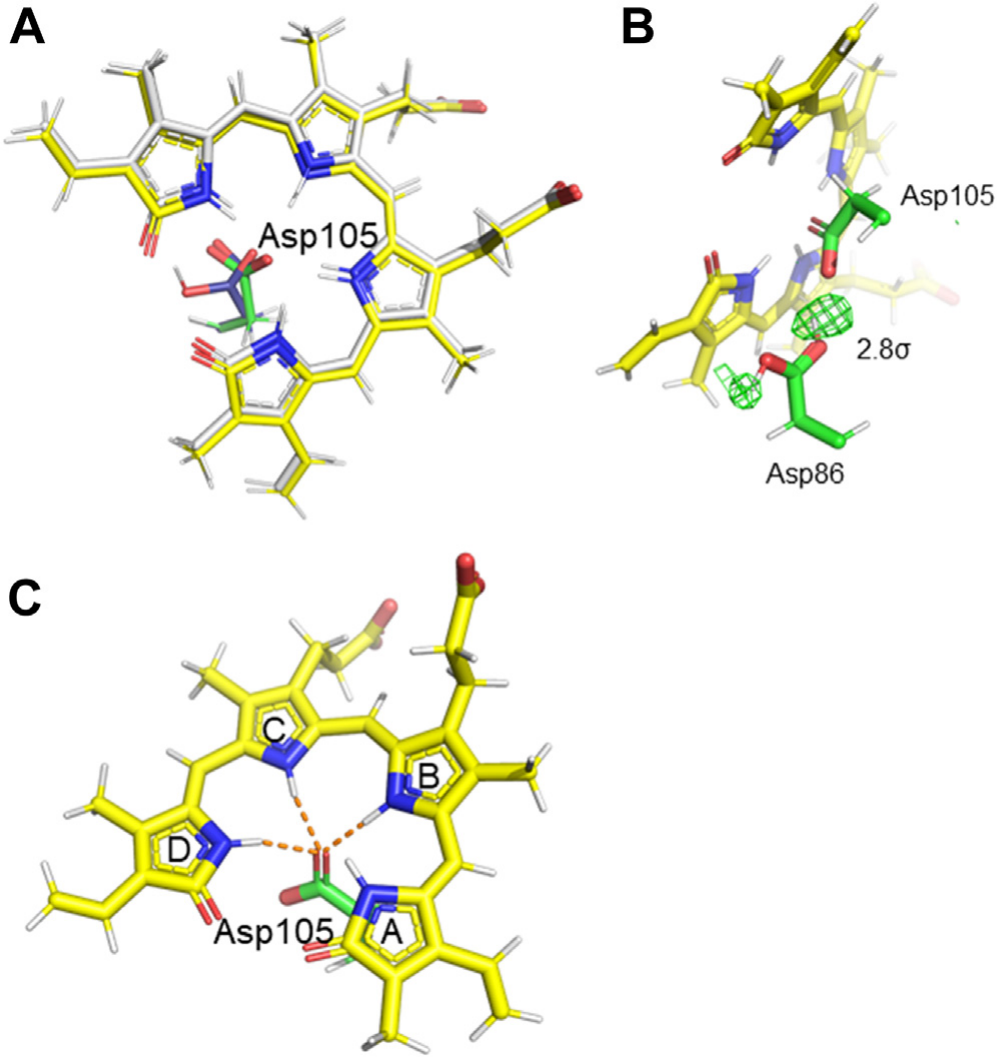
**Structural features near the active site in I86D–BV.** (*A*) Superimposition of I86D–BV and WT–BV. Carbon atoms are colored *white* and *steelblue* for BV and Asp105, respectively, in the WT–BV complex, whereas they are colored yellow and green for BV and Asp105, respectively, in the I86D–BV complex. (*B*) The *F*_o_–*F*_c_ neutron scattering length density omit map (*green* mesh, 2.8σ contour level) and the structural model for Asp86, Asp105, and BV in the I86D–BV complex. The map calculation was performed by omitting the deuterium atoms from the final model. (*C*) Asp105 and hydrogen (deuterium) atoms of BV pyrrole *rings*. The N and O atoms are colored *blue* and *red*, respectively, in all the panels.

Superimposing the structures of the WT–BV and I86D–BV complexes revealed that the conformation of Asp105 in I86D–BV was almost consistent with that of the nonprotonated state of Asp105 (Asp105^−^) in WT–BV (Fig. 4*A*). In the WT–BV complex, Asp105 has two protonation states and two conformations, where the negatively charged Asp105^−^ is considered to correspond to BVH^+^. In fact, neutron crystallographic analysis of I86D–BV showed a residual neutron scattering length density (*F*_o_–*F*_c_ map) near the side chain of Asp105 (near the O atom opposite to BV, Fig. 4*B*). Initially, we considered Asp105 to be protonated in a neutral state; however, after careful analysis, we interpreted this as Asp86 (Ile86 mutated to aspartic acid), followed by Asp105 being protonated. This residual density appears at the midpoint between Asp105 and Asp86, and the protonation of Asp105 or Asp86 is reasonable for structural analysis. Although this neutron scattering density map at 2.1 Å resolution left some ambiguity in the neutron crystallography, it was safe to interpret that Asp86 is protonated based on chemical knowledge (Fig. 4*B*). Therefore, Asp105 is not protonated; moreover, the negatively charged Asp105^−^ interacts with BVH^+^, and the O atom of the Asp105^−^ side chain is hydrogen-bonded to two of the three hydrogen atoms bound to the B-, C-, and D-rings of BVH^+^, with the distances being 1.7 Å, 1.8 Å, and 1.8 Å, respectively (Fig. 4*C*).

Both His88Nδ and His88Nε in I86D–BV were protonated, as seen in WT–BV; similar to WT–BV, but not to D105N–BV, His88 was positively charged in I86D–BV (Fig. S3). His88 also forms a hydrogen bond with the lactam O atom of the BV A-ring. The lactam O atom of the A-ring also forms a hydrogen bond with a water molecule.

### Conformations and protonation states of Glu76 in the mutants

**Figure 5:**
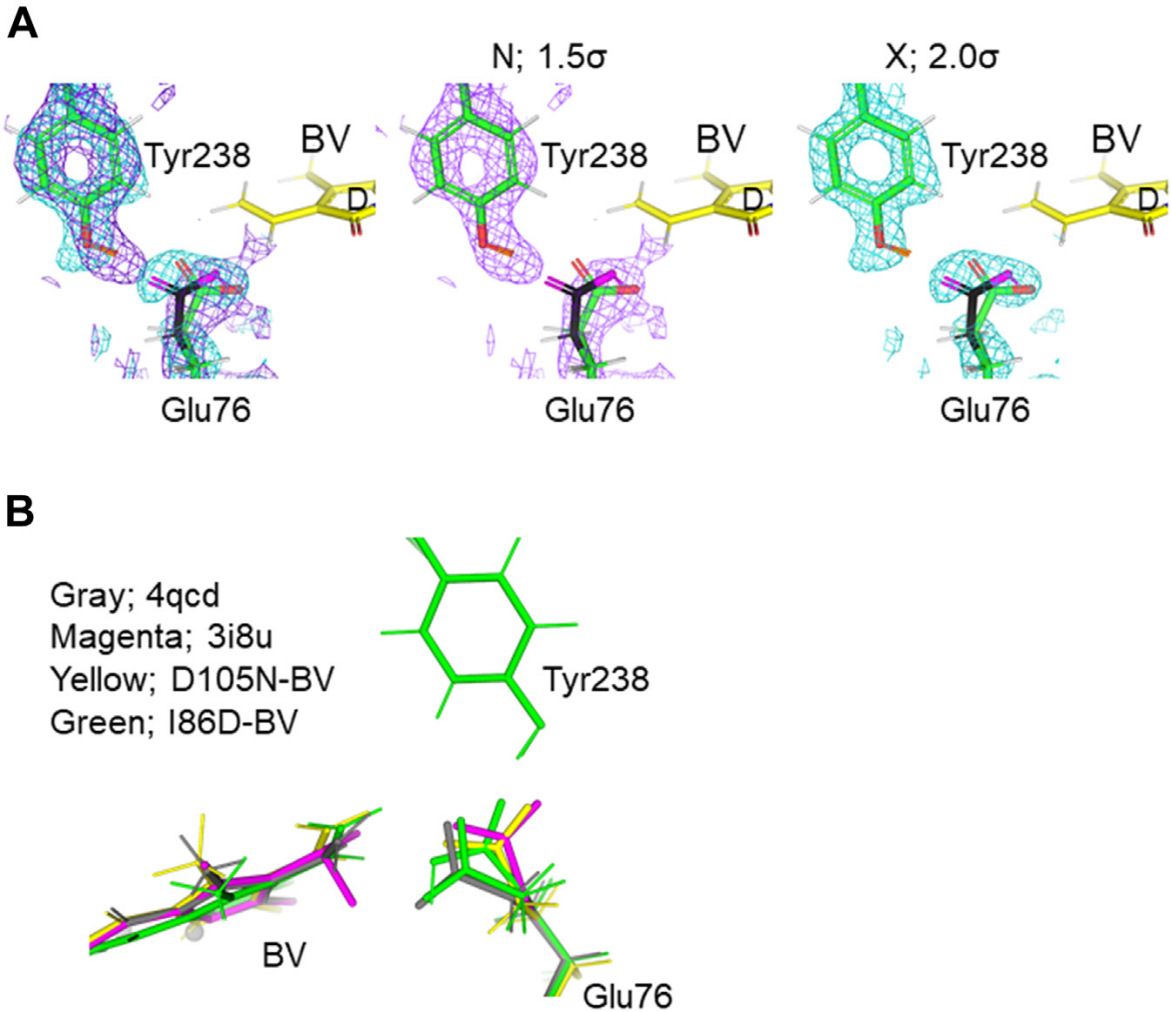
**The Glu76 structures at room temperature.** (*A*) The 2*F*_o_–*F*_c_ neutron scattering density (*purple* mesh, 1.5σ contour level) and electron density (*cyan* mesh, 2.0σ contour level) maps for Glu76 and Tyr238 in I86D–BV, and the structure model. From *left* to *right*: superimposition of both maps, neutron scattering length density map, and electron density map. The structural models are identical. Carbon atoms are colored *yellow* and *green* for BV and amino acid residues, respectively. Carbon atoms of one of the two conformations of Glu76 are colored *black*. The N and O atoms are colored *blue* and *red*, respectively. Hydrogen and deuterium atoms are colored *white* and *orange*, respectively. (*B*) Conformations of Glu76. *Green*, *yellow*, *gray*, and *magenta* sticks represent Glu76 and BV in I86D–BV (this work), D105N–BV (this work), WT–BV (neutron structure, 4QCD) (25), and WT–18EtBV (cryo X-ray structure of a reaction intermediate, 3I8U) (24), respectively. Tyr238 is also shown; the conformation of Tyr238 does not show any differences in all the structures except that the hydrogen atom is not modeled in the cryo X-ray structure of WT–18EtBV.

Glu76 in WT PcyA is considered to be the first proton donor to the substrate BV (24, 25, 30). In the cryo X-ray structure of WT–BV, Glu76 had two conformations (18). Although in the neutron structure of WT–BV at room temperature Glu76 did not show two distinct conformations, the neutron scattering density for this residue was rather low (25). In fact, in the previous neutron crystal structure analysis of WT–BV, there was a difference in the appearance of neutron scattering length density and electron density, which was difficult to interpret. We proposed that, at room temperature, Glu76 was rotating or vibrating around the average position (25). Something similar appeared to be happening in I86D–BV. The neutron scattering length density and electron density maps for Glu76 in I86D–BV did not overlap completely. From both maps, we interpreted I86D–BV as containing both protonated and deprotonated carboxy side chain of Glu76. The protonated Oε of Glu76 was further hydrogen bonded with Tyr238 in I86D–BV (Fig. 5*A*). The conformation of the protonated Glu76 in I86D–BV is similar to that in PcyA WT–18EtBV (PDB ID: 3I8U), an intermediate structure (24). The neutron structure of D105N–BV did not show the protonation of the carboxy side chain of Glu76. In D105N–BV, Glu76 was in a single conformation and hydrogen bonded to Tyr238 (Fig. 5*B*). However, the conformations of Glu76, interacting with Tyr238 in D105N–BV, I86D–BV, and WT–18EtBV, were somewhat different (Fig. 5*B*). Three structures (D105N–BV, I86D–BV, and WT–18EtBV) confirmed that Glu76 interacted with Tyr238. In addition, the side-chain proton (deuteron) of Tyr238 was identified, indicating that the presence of hydrogen bonding was successfully visualized for the first time. Glu76 of I86D–BV is partially oriented toward BV and partially interacts with Tyr238 *via* hydrogen bonding. In contrast, all Glu76 in D105N–BV was oriented toward Tyr238 and formed hydrogen bonds. Neutron crystallography of WT–BV showed that the conformation and protonation state of Glu76 was not clearly determined but the majority of Glu76 was likely to be oriented toward the BV (25), whereas the cryo X-ray structure of WT–18EtBV showed that Glu76 was oriented toward Tyr238 and that it interacted with it through hydrogen bonds (24). These results may indicate the ability to transfer protons, which is likely to be in the order WT > I86D > D105N.

### Protonation states of His88 and the nearby water molecule

In the WT–BV complex, H_3_O^+^ was considered to be present between His88 and His74. However, a later computational study suggested that this interpretation may be incorrect (31, 32).

**Figure 6:**
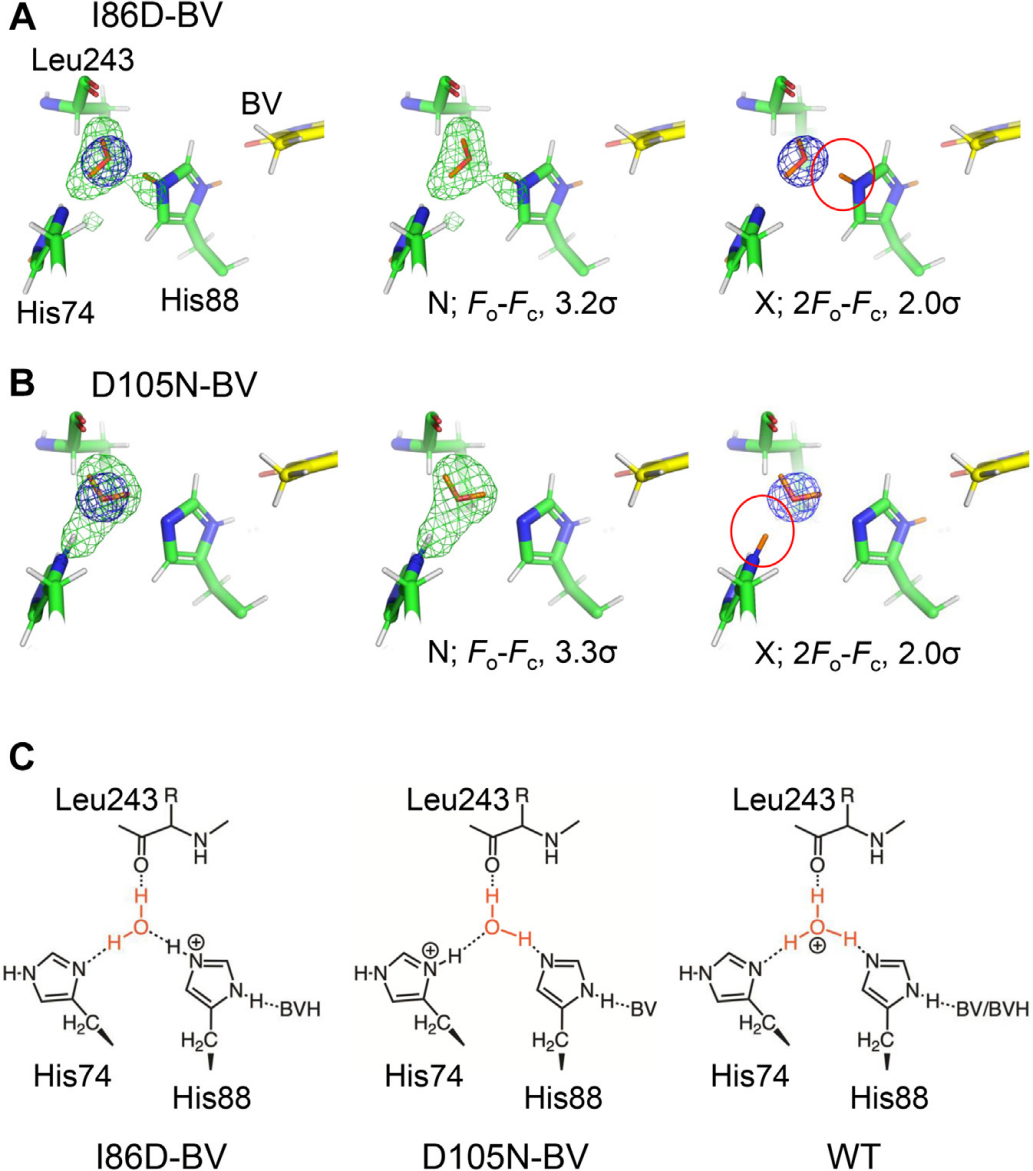
**A key water molecule between His74 and His88.** (*A*) *F*_o_–*F*_c_ neutron scattering length omit map (*green* cage, 3.2σ contour level) and 2*F*_o_–*F*_c_ electron density map (*blue* cage, 2.0σ contour level) between His74 and His88 and their structure models with Leu243 in I86D–BV. From *left* to *right*: superimposition of both maps, neutron scattering length density map, and electron density map. The structural models are identical. (*B*) Corresponding *F*_o_–*F*_c_ neutron scattering length omit map (*green* cage, 3.3σ contour level) and 2*F*_o_–*F*_c_ electron density map (*blue* cage, 2.0σ contour level) and models as (*A*) in D105N–BV. From *left* to *right*: superimposition of both maps, neutron scattering length density map, and electron density map. The structural models are identical. The *F*_o_–*F*_c_ neutron scattering length omit maps were calculated with omitting hydrogen/deuterium atoms but with only O atom at the position of the water molecule. Carbon atoms are colored *yellow* and *green* for BV and amino acid residues, respectively. The N and O atoms are colored *blue* and *red*, respectively. Hydrogen and deuterium atoms are colored white and orange, respectively. (*C*) Chemical structures around the water molecule in I86D–BV, D105N–BV, and WT–BV (25) from *left* to *right*, showing the differences in the orientation of the corresponding water molecule.

In the I86D–BV complex, there was electron density and neutron scattering length density between His88, His74, and Leu243. When only one O atom was modeled in the electron density map, the neutron scattering length density map that extended in three directions remained (Fig. 6*A*). Next, when the hydrogen (deuterium) atoms of the water molecule were placed in the orientation of the Nδ atom of His74 and the main-chain O atom of Leu243, the neutron scattering length density between the O atom of the water molecule and the Nε atom of His88 remained. The residual neutron scattering length density was almost an extension of the imidazole plane of His88. This differs from the previous neutron crystallography of WT–BV, in which the neutron scattering length density map appeared outside the imidazole plane of His88 (25). However, modeling it as H_3_O^+^ and neutral His88 poses no problem for structural analysis. In the present study, the two N atoms of His88 were interpreted as protonated in I86D–BV, based on computational analysis (31, 32).

In the D105N–BV complex, the neutron scattering length density was also observed to extend in three directions when only the O atom of a water molecule was modeled in the electron density map, as observed in I86D–BV (Fig. 6*B*). However, when the deuterium atoms of the water molecule were placed in the direction of His88Nε and the main-chain O atom of Leu243, a neutron scattering length density feature (*F*_o_–*F*_c_) was observed between the O atom of the water molecule and His74Nδ (Fig. 6*B*). The distance between the center of the residual density and the His74Nδ atom was 1.2 Å and that between the center of the residual density and the O atom of the water molecule was 1.5 Å. When the deuterium atom was modeled as binding to His74Nδ, the neutron scattering length density (*F*_o_–*F*_c_) disappeared, indicating that His74Nδ was protonated. It was considered that His74 was positively charged in D105N–BV (Fig. 6*B*). We assumed that His88Nε was protonated, as in the I86D–BV complex, and that the hydrogen (deuterium) atoms of the water molecule were directed toward the main-chain O atom of Leu243 and His74Nδ, resulting in a positive neutron scattering length density around His74Nδ and a negative neutron scattering length density around the O atom of the nearby water molecule (Fig. S4). Based on these observations, it was determined that, in the D105N–BV complex, the hydrogen (deuterium) atoms of the water molecules are oriented toward the main chain O atom of Leu243 and the Nε atom of His88, as well as that His74 is positively charged by proton binding to its Nδ atom. This orientation of the water molecule between His74 and His88 in D105N–BV is very interesting because it is different from that of WT–BV or I86D–BV (Fig. 6*C*). H_3_O^+^ in the WT–BV must be short-lived, and the interpretation of the neutron structure analysis may have been incorrect; however, it is inferred that H_2_O takes on various orientations. In contrast, in the I86D and D105N mutants, this water molecule was fixed with stability, which was not seen in the WT. A comparison of the three structures suggests that, although the water orientation is different in the two mutants, proton transfer is stagnant.

### Computational characterization of UV-Visible spectra by QM/MM method

**Figure 7:**
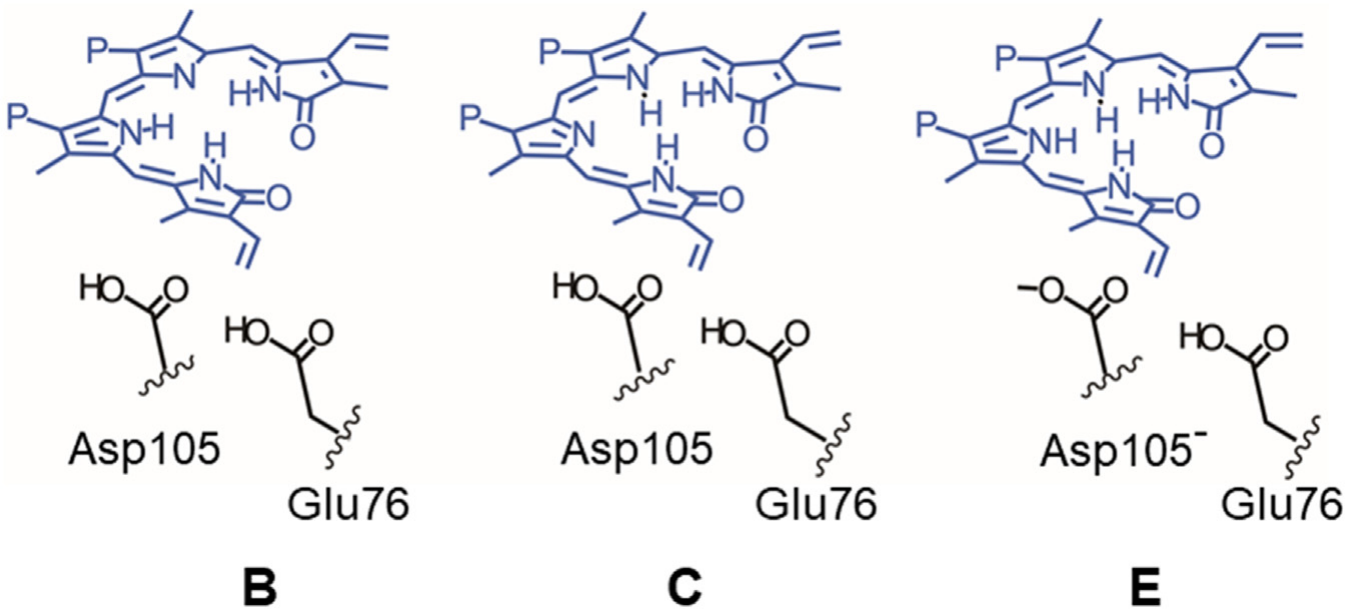
**Protonation states employed in QM/MM calculations and the corresponding labels for each model.** The models B and C represent only the B- and C-pyrrole *rings* that are deprotonated in BV, respectively, whereas Glu76 and Asp105 are protonated. In model E, BV and Glu76 are protonated, while Asp105 is deprotonated.

To discuss the correlations between the BV protonation states and the absorption spectra, we computed the absorption spectra from the experimental neutron structures using the QM/MM method. The initial geometries were the neutron structures of WT PcyA, complexed with BV (PDB ID: 4QCD) (25), I86D–BV, and D105N–BV, which were determined in this work (see Supporting Information for details). The absorption spectra for the three protonation states of BV were computed, as illustrated in Figure 7. The protonation states in which the B- and C-pyrrole rings of BV are deprotonated and Glu76 and Asp105 are protonated are referred to as models B and C, respectively. In model E, all the pyrrole rings of BV and Glu76 were protonated and Asp105 was deprotonated.

**Figure 8:**
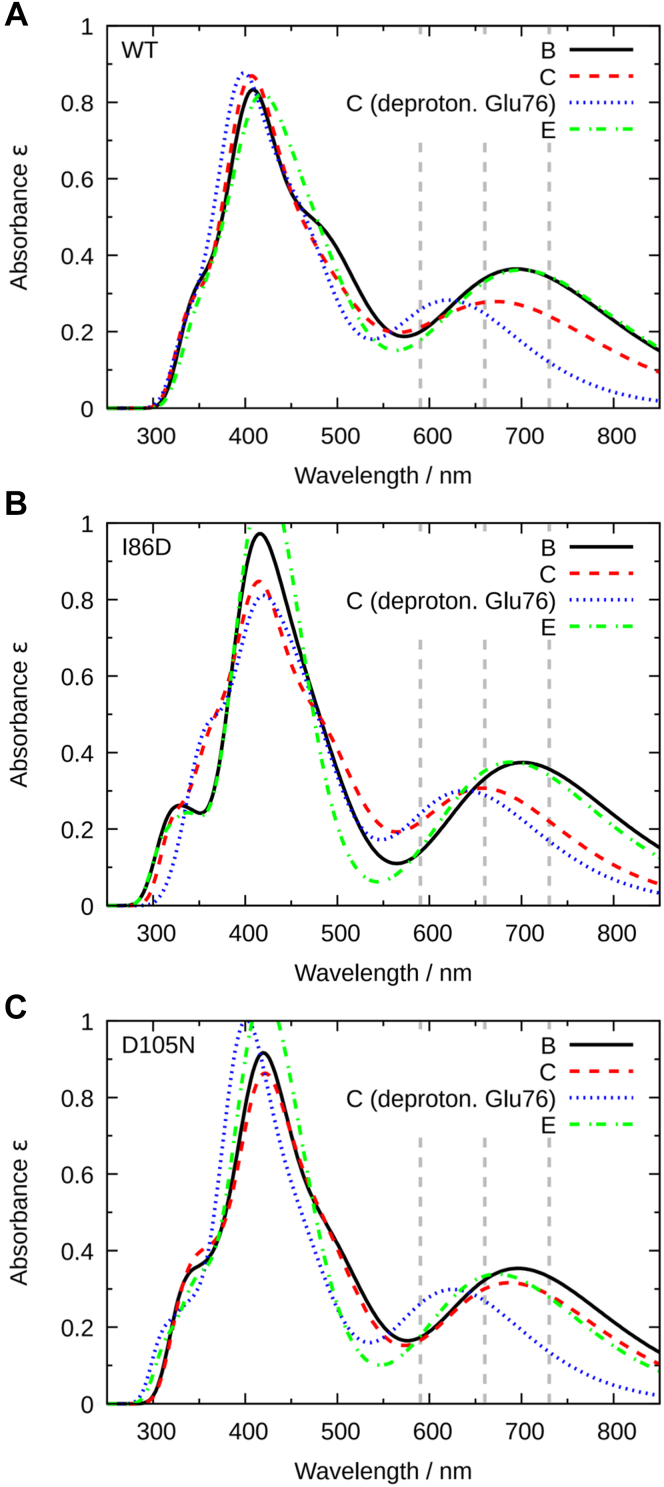
**Computed absorption spectra.** Calculations were performed for the various protonation states shown in Figure 7 for (*A*) WT–BV, (*B*) I86D–BV, and (*C*) D105N–BV, respectively. Spectrum of the C protonation state with Glu76 deprotonated is also shown. Intensities of all spectra are normalized by 50,000 L mol^−1^ cm^−1^. The *gray dashed lines* represent the experimental peak positions at 590 (shoulder peaks of WT–BV and D105N–BV), 660 (main peaks in WT–BV and D105N–BV; second peak in I86D–BV), and 730 nm (main peak in I86D–BV; second peak in WT–BV) (25). The *absorption line* shapes were drawn by GaussView (55).

Figure 8 shows the calculated absorption spectra of WT–BV, I86D–BV, and D105N–BV for the protonation states of BV and Asp105 shown in Figure 7. The peak positions of models B and E in WT (Fig. 8*A*) were between the experimental ones in the Q band at 660 nm (main peaks in WT–BV and D105N–BV, and second peak in I86D–BV) and 730 nm (main peak of I86D–BV, and second peak in WT–BV in the visible region) (25). The C protonation state shows a slightly smaller peak with a shorter wavelength. Furthermore, when the absorption spectrum of the C protonation state with deprotonated Glu76 (blue dotted line) was calculated based on the WT–BV structure, the peak shifted to shorter wavelengths (Fig. 8*A*). Compared with the experimental spectrum of WT–BV (Fig. 1*D*), the protonation states of the active site in WT–BV can be interpreted as a mixture of all protonation states (B, C, C with Glu76 deprotonated, and E).

Figure 8*B* shows the calculated absorption spectra of I86D–BV. The peak centers remained almost the same as those of WT–BV. The computed absorption spectra suggest that the peak shift to a longer wavelength in the Q band of I86D–BV may arise from the predominance of the E state in the neutron structure of I86D–BV, in contrast to the mixture of B, C, C with Glu76 deprotonated, and E in the neutron structure of WT–BV, which has a shorter-wavelength peak. In addition, protonated Glu76 in I86D–BV exists in the neutron structure and is likely to be dominant in solution. Although the experimental peak position at 730 nm of I86D–BV could not be reproduced quantitatively by our QM/MM calculations, possibly due to the inaccuracy of the computational methods such as the time-dependent density-functional theory method, the results qualitatively suggest the dominant existence of BVH^+^ in the I86D–BV complex.

The calculated absorption spectra of D105N–BV showed peak centers similar to the Q band of WT–BV and I86D–BV (Fig. 8*C*). The C protonation state with Glu76 deprotonated in D105N–BV showed a peak shift to a shorter wavelength compared with the other protonation states. This indicated that the C protonation state with Glu76 deprotonated, as found in the neutron structure of D105N–BV, was also dominant in the solution. These findings indicate that the neutron structure of D105N–BV clarifies the presence of deprotonated Glu76, which yields a shorter-wavelength peak in the visible absorption spectra and the possible spectral tuning of the Q band by Glu76.

**Figure 9:**
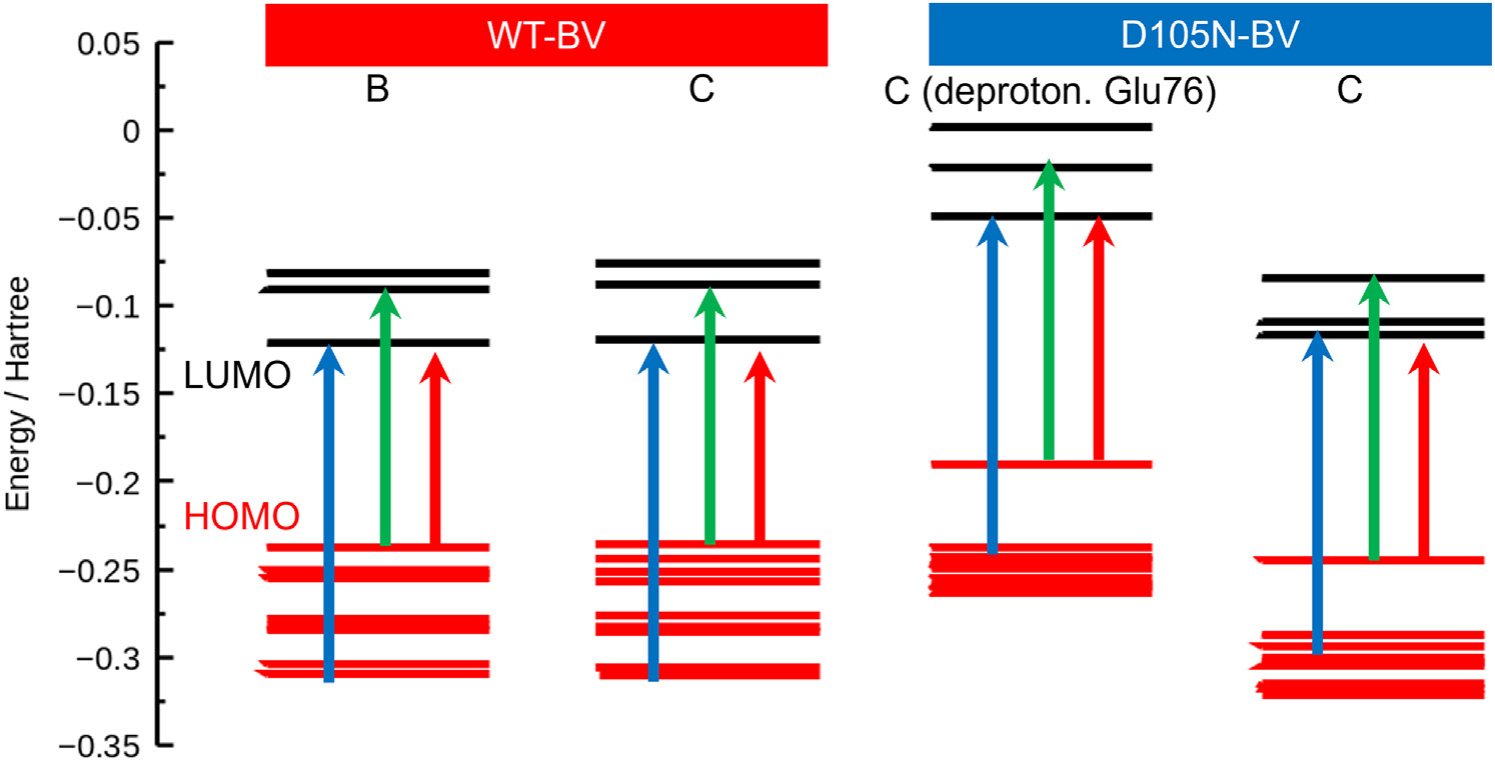
**Energy diagram of WT–BV and D105N–BV for the protonation states shown in**Figure 8**.** The *arrows* schematically denote the most contributing transitions in the three main peaks (See Table S1 for the details of the transitions.). The energy levels represented by *red* and *black lines* are the occupied and unoccupied energy levels, respectively. The figure was made using Gnuplot.

**Figure 10:**
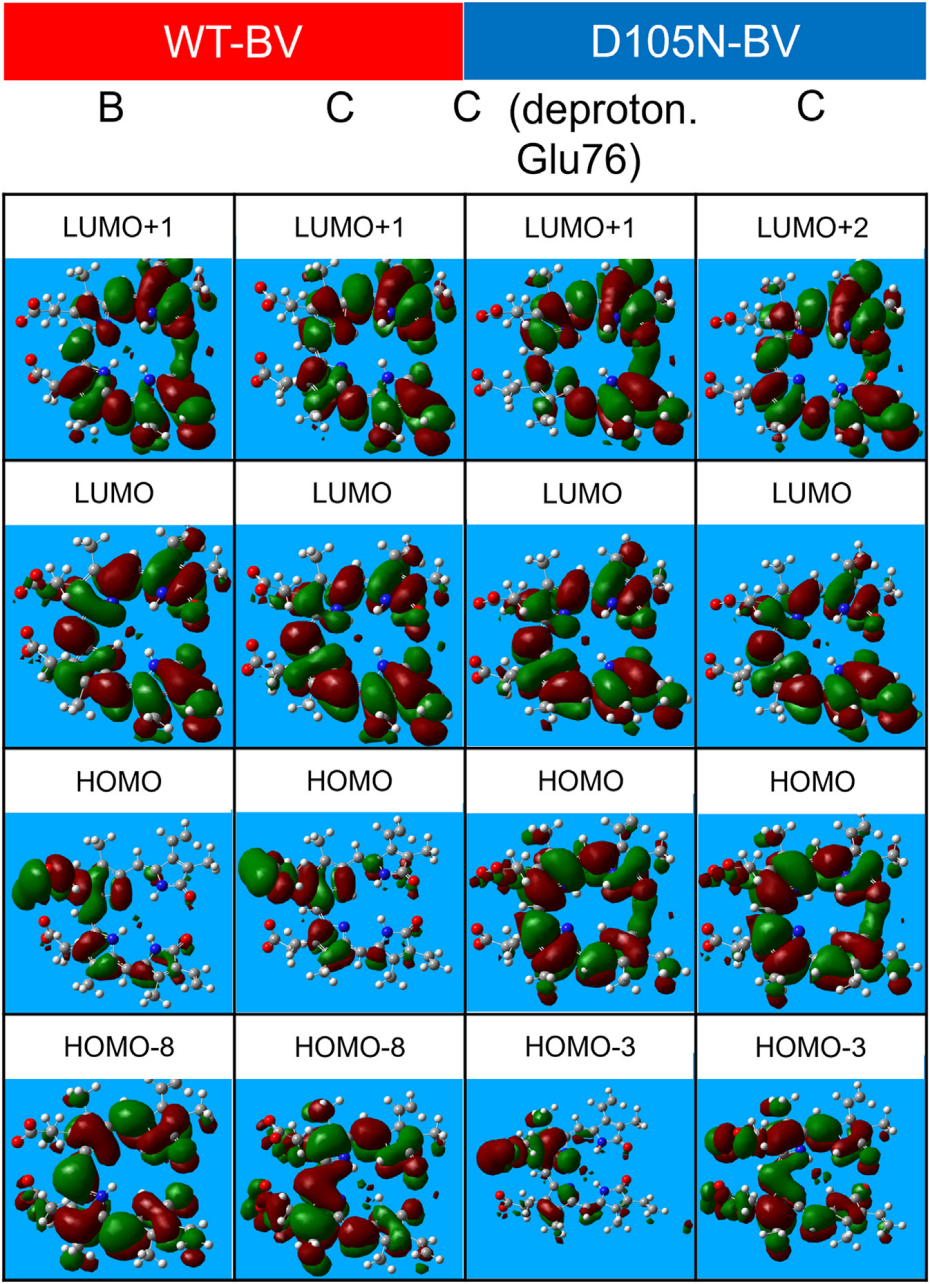
**Kohn−Sham orbitals, the contribution of which is the largest to the electronic transitions in**Figure 9**.** The orbitals were drawn by GaussView (55).

The main peak of the Q band around 700 nm arises mainly from the transition between the highest occupied molecular orbital (HOMO) and the lowest unoccupied molecular orbital (LUMO) of BV, as shown in Figures 9 and 10. In the case of WT–BV, the HOMO of the B and C protonation states has a large contribution from the propionate group connected to the pyrrole B-ring of BV. Therefore, the HOMO of the C protonation state may become slightly more stable, resulting in a larger HOMO–LUMO gap (shorter wavelength). In the C protonation state of D105N–BV, deprotonated Glu76 seems to repel the electron density of the LUMO in the vinyl group and destabilize the orbital. This may lead to a larger HOMO–LUMO gap and a shorter wavelength. Such spectral tuning in proteins by electrostatic interaction with charged amino acids has been observed previously (33). The HOMO of D105N–BV has the characteristic electron density between the distal O atoms of the lactam (C=O) groups in BV because of the stronger hydrogen bond with His88 arising from the mutation, which is found in the experimental neutron structure.

The main peak in the Soret band around 400 nm also arises mainly from the transition between π orbitals of the BV. However, the π orbital in this transition contains more orbitals of the two propionate groups in the BV than in the Q band. These π orbitals were elongated by the two propionate groups to decrease the peak wavelength.

### Hydrogen-bonding network near the substrate BV in the mutants and their activities

The cryo X-ray structure of I86D–BV showed a water molecule between Asp86 and Glu76, with a hydrogen bond linking the two residues. It was inferred that this resulted in the conformation of Glu76, which kept it away from BV and prevented proton transfer from Glu76 to BV (27). However, in the present neuron crystallographic analysis of I86D–BV at room temperature, the electron density for the corresponding water molecule was very low and the neutron scattering length density was almost negligible. It was considered that the water molecule was highly mobile at room temperature, even though it appeared to exist there. In addition, the electron density of this water molecule was clearly visible in the analysis using the data as the X-ray intensity increased (*i.e.*, transmittance increased in the X-ray data collection). Thus, it is proposed that the water molecule is probably trapped when Asp86 and/or Glu76 in its vicinity are reduced by the electrons generated by X-ray irradiation of the crystal. Residual neutron scattering length densities were observed near the carboxy group of Asp86 (Fig. 4*B*), indicating that a proton can bind to either O atom (Oδ1 or Oδ2) of the carboxy group of Asp86. We interpreted Asp86 to have two conformations, *i.e.*, Asp86 is in a neutral state and the protons are bound at different sites: a major component with a proton facing and hydrogen bonding to Asp105 and a minor component facing the opposite direction. In the latter case, the hydrogen bond between Asp105 and Asp86 disappears, but we cannot rule out that some protonated Asp105 mixes and forms a hydrogen bond with Asp86. Nevertheless, it can be said that the majority of Asp105 and Asp86 form hydrogen bonds. The conformation of Asp86 itself was almost identical, regardless of the protonation of the Oδ atom. Since hydrogen atoms are hardly visible in X-ray crystallography, this hydrogen bonding mode between Asp105 and Asp86 was first revealed by neutron crystallographic analysis. Taken together, these findings indicate that even at room temperature, Asp105 is fixed and cannot act as a proton donor and/or acceptor to/from BV, as well as that proton transfer does not occur successfully. Partial protonation of Glu76 was identified for the first time in I86D–BV in this study. Computational analysis during the present study suggested the presence of protonated Glu76 in I86D–BV. However, the proton on the Glu76 carboxy group did not point toward BV; instead, it pointed toward a water molecule between Glu76 and Asp86. Notably, as described above, Glu76 has two conformers: one is protonated and the other is deprotonated. Although Glu76 can form a hydrogen bond with the water molecule between Glu76 and Asp86, the hydrogen (deuterium) atoms of the water molecule could not be identified in this neutron structure. These structural features implied that Glu76 cannot donate protons to BV.

In D105N–BV, Glu76 was completely pointed toward Tyr238 and Glu76 was oriented in the opposite direction to BV. Furthermore, Glu76 in D105N–BV was not protonated, which was confirmed by both neutron structure and computational analysis in this study. Coupled with the inability of Asn105 to participate in proton transfer, the deprotonation and conformation of Glu76 provide additional evidence that D105N is an inactive mutant.

In this study, we analyzed the neutron crystal structures of two PcyA mutants that exhibit characteristic absorption spectra. Based on these neutron structures at hydrogen atom–level resolution, we found that neutral BV was dominant in the D105N mutant, whereas the positively charged state BVH^+^ was dominant in the I86D mutant. In I86D, at room temperature, an electron is likely to easily move to BVH^+^, generating a small amount of BVH radical species, thereby resulting in a mixture of different Asp86 protonation sites, the disappearance of a water molecule connecting Asp105 and Asp86, and a mixture of two conformations and protonation states of Glu76 in I86D. These two mutants had a fixed water molecule between His74 and His86 in the vicinity of the substrate BV. The orientation of this water molecule was fixed in these mutants, whereas the water molecule had dual orientations (or hydronium ion is present) in the WT. The difference in the water molecule stability appears to correlate with the protonation states of BV and enzyme activity.

One of the most significant results of this study was the visualization of the correlation between the protonation states of BV, the catalytic residue Glu76, and the absorption spectra of the PcyA–BV complex. A single hydrogen atom was found to slightly alter the electronic state and conformation of BV, while also significantly altering the absorption spectrum of the PcyA–BV complex. QM/MM calculations based on the structure obtained by neutron crystallography suggest that the protonated state of Glu76 is involved not only in the enzymatic reaction but also in the absorption spectrum of the PcyA–BV complex.

The protonation state of the substrate was controlled by mutating a single amino acid residue and altering the absorption spectrum of the PcyA–BV complex. Further studies are expected to lead to the controlling of the reaction and to the development of novel products that absorb light of various energies.

## Experimental procedures

### Expression and purification of D105N and I86D

Site-directed mutagenesis of *Synechocystis* PcyA was performed with the QuikChange Site-Directed Mutagenesis Kit (Agilent Technology), using plasmid pET21a-*pcyA* as the template (18). Two oligonucleotides (5′-CCTTTGTTTGGCTGTAACATTGTGGCCGGCC-3′ and 5′-GGCCGGCCACAATGTTACAGCCAAACAAAGG-3′) were used to introduce the D105N mutation into *pcyA*. Sequence analysis verified that the construct was error-free. The procedures for the I86D mutation have been reported previously (27). The D105N and I86D mutant proteins were expressed and purified as previously described for the WT and mutant PcyA (18, 27). Since deuterated reagents were not used in the purification, the resulting samples were in hydrogenated forms.

### Crystallizations of the D105N–BV and I86D–BV complex for neutron crystallography

The crystallization procedures and conditions for D105N–BV were slightly modified from a previously reported method to grow larger crystals suitable for neutron crystallography (25, 34). For D105N–BV, the complex solution was prepared by mixing a molar ratio of D105N:BV = 1:1.1, after which crystallization was performed. The sitting drop vapor diffusion method was used for crystallization, and the drop volume and reservoir solution volume were 50 and 1500 μl, respectively. The reservoir solution contained 200 mM NaCl, 1.2 M ammonium sulfate, and 50 mM MES (pH 6.3). For I86D–BV, the crystallization conditions and procedures were followed as reported in a previous paper (34). Crystallization procedures for both D105N–BV and I86D–BV were performed under dark conditions, except for a weak green spotlight. The crystallization plates were wrapped in aluminum foil to shield them from light and were placed in an incubator at 293 K. Falcon 24-well cell culture plates (Corning) were used as crystallization plates.

### Neutron diffraction experiments

Deuterium exchange of exchangeable hydrogen atoms in the crystals was performed prior to the neutron diffraction experiments. For the D105N–BV crystals, the crystals were soaked in the deuterated buffer (1.2 M ammonium sulfate-*d*_8_ [98%; Cambridge Isotope Laboratories], 50 mM MES [pD 6.7], 200 mM NaCl) and the solution was replaced after 2, 6, 11, and 16 days, for a total of 21 days. As previously reported, a similar procedure was applied for the crystal of I86D–BV (34). The I86D–BV crystal was soaked in deuterated crystallization solution, and the solution was replaced after 5 days, 1 week, and 2 weeks, for a total of 45 days. The soaked crystals of D105N–BV and I86D–BV were mounted in quartz glass capillaries with 3.5 mm φ and 0.01 mm thickness and with 2.5 mm φ and 0.01 mm thickness, respectively.

Time-of-flight neutron diffraction data of the D105N–BV crystal were collected at BL03, IBARAKI biological crystal diffractometer iBIX (35, 36) at the Materials and Life Sciences Experimental Facility (MLF) of the Japan Proton Accelerator Research Complex (J-PARC, Tokai, Japan) at approximately 300 K, under dark conditions. Thirty-four wavelength-shifting fiber-based scintillator neutron detectors with a sensitive area of 133 × 133 mm^2^ were used to collect the data. A total of 30 datasets were collected using a wavelength of 2.63 − 6.06 Å, with a detector distance of 492 mm. The exposure time for each dataset was 6 h 40 min at 600 kW. Time-of-flight neutron data were indexed, integrated, scaled, and processed using STARGazer (37, 38).

Neutron diffraction data of the I86D–BV crystal were collected at the BIODIFF beamline (MLZ, FRM II, Garching, Germany) (39), where a monochromatic neutron beam with a wavelength of 3.1 Å was used. The exposure time and rotation range per frame were 65 min and 0.3°, respectively, and the detector-to-sample distance was 200 mm. In total, 228 frames were collected at approximately 300 K. The monochromatic neutron data were indexed, integrated, scaled, and processed using the program package HKL2000 (40).

During data collection at both J-PARC and FRM II, the light was turned off and the samples were covered with aluminum foil to avoid exposing the crystals to visible light. These large D105N–BV and I86D–BV crystals were used for subsequent X-ray diffraction experiments at approximately 300 K. The statistics for the two neutron diffraction datasets are presented in Table 2.

**Table 2:** Statistics for neutron and X-ray diffraction data for the crystals of D105N–BV and I86D–BV at 24 °C

Diffraction data collection conditions				
	D105N–BV		I86D–BV	
Sources	Neutron	X-ray	Neutron	X-ray
Beamline	J-PARC, MLF, BL03 iBIX	PF-AR, NW12A	MLZ, FRM II, BIODIFF	PF, BL-17A
Transmittance (%)	100	10	100	1
Slit size	3.0 mm φ	0.20 mm (vertical) × 0.13 mm (horizontal)	2.5 mm φ	0.02 mm × (vertical) 0.02 mm (horizontal)
Wavelength (Å)	2.63−6.06	1.0	3.1	1.0
Oscillation range (º)	still	1.0	0.3	1.0
Exposure time	6 h 40 min/set	0.5 s/1.0º	65 min/0.3º	1.0 s/1.0º
Collected images	30 sets	135º	68.4º	135º
Data statistics				
	D105N–BV		I86D–BV	
Space group	*P*2_1_2_1_2			
Cell dimensions (Å)	*a* = 71.17, *b* = 97.52, *c* = 43.28		*a* = 71.26, *b* = 97.45, *c* = 43.10	
Sources	Neutron	X-ray	Neutron	X-ray
Resolution range (Å)	21.16–2.10 (2.17–2.10)	42.28–1.38 (1.40–1.38)	55.00–2.00 (2.05–2.00)	50.00–1.90 (1.93–1.90)
I/σI	9.18 (1.79)	12.2 (2.10)	6.53 (1.82)	18.71 (2.50)
Completeness (%)	99.1 (99.3)	99.6 (100.0)	84.2 (77.8)	100.0 (100.0)
Observed reflections	125,974	303,015	34,563	118,865
Unique reflections	18,158	62,402	17,734	24,473
Redundancy	6.9 (5.4)	4.9 (4.5)	1.9 (1.7)	5.0 (4.9)
*R*_sym_	0.239 (0.978)	0.068 (0.637)	0.095 (0.351)	0.085 (0.540)
*R*_pim_	0.096 (0.449)	0.034 (0.038)	0.075 (0.254)	0.043 (0.268)
*CC*_1/2_	0.983 (0.479)	0.997 (0.752)	0.887 (0.762)	0.988 (0.797)

### X-ray diffraction experiments

X-ray diffraction data from the same D105N–BV crystal used for neutron diffraction were collected using a Dectris Pilatus3 S2M pixel detector at NW12A at the Photon Factory Advanced Ring (PF-AR; Tsukuba, Japan) at room temperature. The wavelength of synchrotron radiation, transmittance, and slit size were 1.0 Å, 10%, and 0.2 mm (horizontal) × 0.13 mm (vertical), respectively. The sample-to-detector distance, oscillation range, and exposure time were 200 mm, 1.0°, and 0.5 s, respectively. To minimize the amount of damage caused by radiation, the position of the crystal during irradiation was changed for each shot (41). A total of 135 images were collected, following which data were integrated, merged, and processed using XDS (42).

X-ray diffraction data from the same I86D–BV crystal used for neutron diffraction were collected using a Dectris Pilatus3 6M pixel detector at BL-17A at the Photon Factory (PF; Tsukuba, Japan) at room temperature. The wavelength of the synchrotron radiation, transmittance, and slit size were 1.0 Å, 1%, and 0.02 mm × 0.02 mm, respectively. The sample-to-detector distance, oscillation range, and exposure time were 250 mm, 1.0°, and 1.0 s, respectively. To minimize the amount of damage due to radiation, the position of the crystal during irradiation was changed for each shot (41). A total of 135 images for each X-ray intensity data were collected. The data were integrated, merged, and processed using HKL2000 (40).

The neutron and X-ray diffraction data statistics are listed in Table 2.

### Structure refinements

The neutron structure of D105N–BV was determined using the “cryo” D105N–BV complex structure (PDB ID: 3F0L) (29), from which water molecules and BV were removed as the initial model. The first refinement was performed using the rigid-body refinement program, Refmac5 (43, 44), with only X-ray diffraction data collected at room temperature. The structure model was modified using the graphics program COOT (45, 46), by modifying the coordinates and conformations of amino acid residues to fit the electron density map, adding multiconformations of some side chains and adding or deleting water molecules; these modifications were made until the *R* and *R*_free_ values converged.

Subsequently, a joint refinement with both neutron and X-ray data was carried out using Phenix (47). The 2.10-Å-resolution neutron data and the 1.38-Å-resolution X-ray data were used for structure refinement. Five percent of the data was selected using Phenix for cross-validation. The structure refinements were repeated multiple times with phenix_refine, while modifying the model using COOT.

Hydrogen or deuterium atoms were then added to the hydrogen/deuterium exchangeable sites on the main and side chains of the amino acid residues of the model using Phenix.ready_set, hydrogen/deuterium atoms were modified again using COOT, hydrogen/deuterium exchange of the water molecules was performed using Phenix.ready_set, and the model was further modified multiple times using COOT. The orientations of the water molecules were manually adjusted by observing both the neutron scattering length density map and the electron density map that was calculated before including hydrogen/deuterium in COOT.

Furthermore, to determine the protonation state of BV in the D105N–BV complex, the occupancies of the deuterium atoms bound to the pyrrole N atoms of the A-, B-, and D-rings of BV, where the neutron scattering length density was observed, were calculated; the occupancies of the deuterium atoms bound to the A- and D-rings were fixed at 1.0. To refine the temperature factors for six sets, the B-ring deuterium occupancy was varied, in steps of 0.1, from 0.5 to 1.0.

The neutron structure of I86D–BV was determined similar to that of D105N–BV neutron structure determination. The initial phases were determined *via* the molecular replacement method using the high-resolution cryo X-ray structure of the I86D–BV complex (PDB ID: 5B4H) (27). The 2.00-Å-resolution neutron data and 1.90-Å-resolution X-ray data were used for the joint refinement. The occupancies of the deuterium atoms bound to the pyrrole N atoms of the A-, B-, C-, and D-rings of BV, where the neutron scattering length density was observed, were calculated (varying by 0.1) to refine the temperature factors of deuterium atoms in BV.

The refinement statistics are presented in Table 1. Figures 1*B*, 2, *A*, *C* and *D*, 3, *A* and *B*, Figure 4, Figure 5, Figure 6, *A* and *B* were produced using PyMOL (Schrödinger L, DeLano W. PyMOL. 2020. http://www.pymol.org/pymol). The atomic coordinates and structure factors for the neutron structures of the PcyA D105N–BV complex and I86D–BV complex at room temperature have been deposited at the Protein Data Bank Japan (https://pdbj.org/). The PDB IDs for D105N–BV and I86D–BV are 7YKB and 7YK9, respectively.

### Computational methods

Detailed procedures for calculating the absorption spectra, to discuss the correlations between the neutron structures and absorption spectra of the PcyA mutants in complexes with BV, are described in the supporting information (Figs. S5–S8 and Table S1).

## Data availability

The structure presented in this study is available in the PDB under the codes 7YKB and 7YK9. All remaining data are contained within the published article.

## Supporting information

This article contains supporting information (48, 49, 50, 51, 52, 53, 54, 55, 56, 57, 58, 59, 60).

## Conflict of interest

The authors declare that they have no conflicts of interest with the contents of this article.

## References

[1] Rhie G., Beale S.I. (1992). Biosynthesis of phycobilins. Ferredoxin-supported nadph-independent heme oxygenase and phycobilin-forming activities from Cyanidium caldarium. J. Biol. Chem..

[2] Yamanaka G., Lundell D.J., Glazer A.N. (1982). Molecular architecture of a light-harvesting antenna. Isolation and characterization of phycobilisome subassembly particles. J. Biol. Chem..

[3] Watanabe M., Ikeuchi M. (2013). Phycobilisome: architecture of a light-harvesting supercomplex. Photosynth Res..

[4] Schafer E., Bowle C. (2002). Phytochrome-mediated photoperception and signal transduction in higher plants. EMBO Rep..

[5] Rockwell N.C., Martin S.S., Lagarias J.C. (2016). Identification of cyanobacteriochromes detecting Far-red light. Biochemistry.

[6] Rockwell N.C., Martin S.S., Lagarias J.C. (2017). There and back again: loss and reacquisition of two-Cys Photocycles in cyanobacteriochromes. Photochem. Photobiol..

[7] Narikawa R., Nakajima T., Aono Y., Fushimi K., Enomoto G., Ni Ni W. (2015). A biliverdin-binding cyanobacteriochrome from the chlorophyll d-bearing cyanobacterium Acaryochloris marina. Sci. Rep..

[8] Bhoo S.H., Davis S.J., Walker J., Karniol B., Vierstra R.D. (2001). Bacteriophytochromes are photochromic histidine kinases using a biliverdin chromophore. Nature.

[9] Hirose Y., Rockwell N.C., Nishiyama K., Narikawa R., Ukaji Y., Inomata K. (2013). Green/red cyanobacteriochromes regulate complementary chromatic acclimation via a protochromic photocycle. Proc. Natl. Acad. Sci. U. S. A..

[10] Nagae T., Unno M., Koizumi T., Miyanoiri Y., Fujisawa T., Masui K. (2021). Structural basis of the protochromic green/red photocycle of the chromatic acclimation sensor RcaE. Proc. Natl. Acad. Sci. U. S. A..

[11] Sugishima M., Wada K., Unno M., Fukuyama K. (2019). Bilin-metabolizing enzymes: site-specific reductions catalyzed by two different type of enzymes. Curr. Opin. Struct. Biol..

[12] Frankenberg N., Mukougawa K., Kohchi T., Lagarias J.C. (2001). Functional genomic analysis of the HY2 family of ferredoxin-dependent bilin reductases from oxygenic photosynthetic organisms. Plant Cell.

[13] Kohchi T., Mukougawa K., Frankenberg N., Masuda M., Yokota A., Lagarias J.C. (2001). The Arabidopsis HY2 gene encodes phytochromobilin synthase, a ferredoxin-dependent biliverdin reductase. Plant Cell.

[14] Frankenberg N., Lagarias J.C. (2003). Phycocyanobilin:ferredoxin oxidoreductase of Anabaena sp. PCC 7120. Biochemical and spectroscopic. J. Biol. Chem..

[15] Hughes J., Lamparter T. (1999). Prokaryotes and phytochrome. The connection to chromophores and signaling. Plant Physiol..

[16] Unno M., Sugishima M., Wada K., Fukuyama K., Akitsu T. (2013). Integrating Approach Photofunctional Hybrid Materials for Energy and the Environment.

[17] Chen Y.R., Su Y.S., Tu S.L. (2012). Distinct phytochrome actions in nonvascular plants revealed by targeted inactivation of phytobilin biosynthesis. Proc. Natl. Acad. Sci. U. S. A..

[18] Hagiwara Y., Sugishima M., Takahashi Y., Fukuyama K. (2006). Crystal structure of phycocyanobilin:ferredoxin oxidoreductase in complex with biliverdin IXalpha, a key enzyme in the biosynthesis of phycocyanobilin. Proc. Natl. Acad. Sci. U. S. A..

[19] Sugishima M., Wada K., Fukuyama K., Yamamoto K. (2020). Crystal structure of phytochromobilin synthase in complex with biliverdin IXalpha, a key enzyme in the biosynthesis of phytochrome. J. Biol. Chem..

[20] Busch A.W., Reijerse E.J., Lubitz W., Frankenberg-Dinkel N., Hofmann E. (2011). Structural and mechanistic insight into the ferredoxin-mediated two-electron reduction of bilins. Biochem. J..

[21] Dammeyer T., Hofmann E., Frankenberg-Dinkel N. (2008). Phycoerythrobilin synthase (PebS) of a marine virus. Crystal structures of the biliverdin complex and the substrate-free form. J. Biol. Chem..

[22] Dammeyer T., Frankenberg-Dinkel N. (2006). Insights into phycoerythrobilin biosynthesis point toward metabolic channeling. J. Biol. Chem..

[23] Tu S.L., Sughrue W., Britt R.D., Lagarias J.C. (2006). A conserved histidine-aspartate pair is required for exovinyl reduction of biliverdin by a cyanobacterial phycocyanobilin:ferredoxin oxidoreductase. J. Biol. Chem..

[24] Hagiwara Y., Sugishima M., Khawn H., Kinoshita H., Inomata K., Shang L. (2010). Structural insights into vinyl reduction regiospecificity of phycocyanobilin:ferredoxin oxidoreductase (PcyA). J. Biol. Chem..

[25] Unno M., Ishikawa-Suto K., Kusaka K., Tamada T., Hagiwara Y., Sugishima M. (2015). Insights into the proton transfer mechanism of a bilin reductase PcyA following neutron crystallography. J. Am. Chem. Soc..

[26] Stoll S., Gunn A., Brynda M., Sughrue W., Kohler A.C., Ozarowski A. (2009). Structure of the biliverdin radical intermediate in phycocyanobilin:ferredoxin oxidoreductase identified by high-field EPR and DFT. J. Am. Chem. Soc..

[27] Hagiwara Y., Wada K., Irikawa T., Sato H., Unno M., Yamamoto K. (2016). Atomic-resolution structure of the phycocyanobilin:ferredoxin oxidoreductase I86D mutant in complex with fully protonated biliverdin. FEBS Lett..

[28] Tu S.L., Gunn A., Toney M.D., Britt R.D., Lagarias J.C. (2004). Biliverdin reduction by cyanobacterial phycocyanobilin:ferredoxin oxidoreductase (PcyA) proceeds via linear tetrapyrrole radical intermediates. J. Am. Chem. Soc..

[29] Kohler A.C., Gae D.D., Richley M.A., Stoll S., Gunn A., Lim S. (2010). Structural basis for hydration dynamics in radical stabilization of bilin reductase mutants. Biochemistry.

[30] Tu S.L., Rockwell N.C., Lagarias J.C., Fisher A.J. (2007). Insight into the radical mechanism of phycocyanobilin-ferredoxin oxidoreductase (PcyA) revealed by X-ray crystallography and biochemical measurements. Biochemistry.

[31] Ikeda T., Saito K., Hasegawa R., Ishikita H. (2017). The existence of an Isolated hydronium ion in the Interior of proteins. Angew. Chem. Int. Ed. Engl..

[32] Iijima E., Gleeson M.P., Unno M., Mori S. (2018). QM/MM Investigation for protonation states in a bilin reductase PcyA-biliverdin IXɑ complex. Chemphyschem.

[33] Song C., Narikawa R., Ikeuchi M., Gärtner W., Matysik J. (2015). Color tuning in red/green cyanobacteriochrome AnPixJ: photoisomerization at C15 causes an excited-state destabilization. J. Phys. Chem. B.

[34] Igarashi K., Hagiwara Y., Sugishima M., Wada K., Fukuyama K., Ikeda A. (2018). Crystal growth of a bilin reductase PcyA I86D mutant−substrate complex for neutron crystallography. Cryst. Growth Des..

[35] Tanaka I., Kusaka K., Hosoya T., Niimura N., Ohhara T., Kurihara K. (2010). Neutron structure analysis using the IBARAKI biological crystal diffractometer (iBIX) at J-PARC. Acta Crystallogr. D Biol. Crystallogr..

[36] Kusaka K., Hosoya T., Yamada T., Tomoyori K., Ohhara T., Katagiri M. (2013). Evaluation of performance for IBARAKI biological crystal diffractometer iBIX with new detectors. J. Synchrotron Radiat..

[37] Yano N., Yamada T., Hosoya T., Ohhara T., Tanaka I., Niimura N. (2018). Status of the neutron time-of-flight single-crystal diffraction data-processing software STARGazer. Acta Crystallogr. D Struct. Biol..

[38] Ohhara T., Kusaka K., Hosoya T., Kurihara K., Tomoyori K., Niimura N. (2009). Development of data processing software for a new TOF single crystal neutron diffractometer at J-PARC. Nucl. Instr. Methods Phys. Res. A.

[39] Ostermann A., Schrader T. (2015). BIODIFF: diffractometer for large unit cells. J. Large-Scale Res. Facil..

[40] Otwinowski Z., Minor W. (1997). Processing of X-ray diffraction data collected in oscillation mode. Methods Enzymol..

[41] Zeldin O.B., Brockhauser S., Bremridge J., Holton J.M., Garman E.F. (2013). Predicting the X-ray lifetime of protein crystals. Proc. Natl. Acad. Sci. U. S. A..

[42] Kabsch W. (2010). Xds. Acta Crystallogr. D Biol. Crystallogr..

[43] Murshudov G.N., Skubak P., Lebedev A.A., Pannu N.S., Steiner R.A., Nicholls R.A. (2011). REFMAC5 for the refinement of macromolecular crystal structures. Acta Crystallogr. D Biol. Crystallogr..

[44] Murshudov G.N., Vagin A.A., Dodson E.J. (1997). Refinement of macromolecular structures by the maximum-likelihood method. Acta Crystallogr. D Biol. Crystallogr..

[45] Emsley P., Cowtan K. (2004). Coot: model-building tools for molecular graphics. Acta Crystallogr. D Biol. Crystallogr..

[46] Emsley P., Lohkamp B., Scott W.G., Cowtan K. (2010). Features and development of coot. Acta Crystallogr. D Biol. Crystallogr..

[47] Liebschner D., Afonine P.V., Baker M.L., Bunkoczi G., Chen V.B., Croll T.I. (2019). Macromolecular structure determination using X-rays, neutrons and electrons: recent developments in phenix. Acta Crystallogr. D Struct. Biol..

[48] Wang J., Wang W., Kollman P.A., Case D.A. (2006). Automatic atom type and bond type perception in molecular mechanical calculations. J. Mol. Graph. Model..

[49] Vreven T., Byun K.S., Komáromi I., Dapprich S., Montgomery J.A., Morokuma K. (2006). Combining quantum mechanics methods with molecular mechanics methods in ONIOM. J. Chem. Theory Comput..

[50] Chung L.W., Sameera W.M.C., Ramozzi R., Page A.J., Hatanaka M., Petrova G.P. (2015). The ONIOM method and its applications. Chem. Rev..

[51] Frisch M.J., Trucks G.W., Schlegel H.B., Scuseria G.E., Robb M.A., Cheeseman J.R. (2016).

[52] Zhao Y., Truhlar D.G. (2008). The M06 suite of density functionals for main group thermochemistry, thermochemical kinetics, noncovalent interactions, excited states, and transition elements: two new functionals and systematic testing of four M06-class functionals and 12 other functionals. Theor. Chem. Acc..

[53] Cornell W.D., Cieplak P., Bayly C.I., Gould I.R., Merz K.M., Ferguson D.M. (1995). A second generation force field for the simulation of proteins, nucleic acids, and organic molecules. J. Am. Chem. Soc..

[54] Adamo C., Jacquemin D. (2013). The calculations of excited-state properties with time-dependent density functional theory. Chem. Soc. Rev..

[55] Roy D., Todd A.K., John M.M. (2016).

[56] Yanai T., Tew D.P., Handy N.C. (2004). A new hybrid exchange–correlation functional using the Coulomb-attenuating method (CAM-B3LYP). Chem. Phys. Lett..

[57] Becke A.D. (1993). Density-functional thermochemistry. III. The role of exact exchange. J. Chem. Phys..

[58] Adamo C., Barone V. (1999). Toward reliable density functional methods without adjustable parameters: the PBE0 model. J. Chem. Phys..

[59] Henderson T.M., Izmaylov A.F., Scalmani G., Scuseria G.E. (2009). Can short-range hybrids describe long-range-dependent properties?. J. Chem. Phys..

[60] Schäfer A., Horn H.W., Ahlrichs R. (1992). Fully optimized contracted Gaussian basis sets for atoms Li to Kr. J. Chem. Phys..

