# Supplementary material for: Neutron crystallography and quantum chemical analysis of bilin reductase PcyA mutants reveal substrate and catalytic residue protonation states
Source: J Biol Chem. 2022 Dec 1;299(1):102763. doi: 10.1016/j.jbc.2022.102763 (PMC9800206; doi:10.1016/j.jbc.2022.102763)
Supplement: Supplemental Figures S1–S8 and Table S1 [file mmc1.docx]

**Supporting information for**

**Neutron crystallography and quantum chemical analysis of bilin reductase PcyA mutants reveal substrate and catalytic residue protonation states**

**Tatsuya Joutsuka^1, 2, ‡,^ *, Ryota Nanasawa^1, ‡^, Keisuke Igarashi^1, ‡^, Kazuki Horie^1^, Masakazu Sugishima^3^, Yoshinori Hagiwara^4^, Kei Wada^5^, Keiichi Fukuyama^6^, Naomine Yano^2^, Seiji Mori^1, 2^, Andreas Ostermann^7^, Katsuhiro Kusaka^2^, and Masaki Unno^1, 2,^ ***

From the ^1^Graduate School of Science and Engineering, Ibaraki University, 4-12-1 Nakanarusawa, Hitachi, Ibaraki 316-8511, Japan; ^2^Frontier Research Center for Applied Atomic Sciences, Ibaraki University, 162-1 Shirakata, Naka-Tokai, Ibaraki 319-1106, Japan; ^3^Department of Medical Biochemistry, Kurume University School of Medicine, 67 Asahimachi, Kurume, Fukuoka 830-0011, Japan; ^4^Department of Biochemistry and Applied Chemistry, National Institute of Technology, Kurume College, 1-1-1 Komorino, Kurume, Fukuoka 830-8555, Japan; ^5^Department of Medical Sciences, University of Miyazaki, 5200 Kihara, Kiyotake, Miyazaki, Miyazaki 889-1692, Japan; ^6^Graduate School of Science, Osaka University, 1-1 Machikaneyama, Toyonaka, Osaka 560-0043, Japan; ^7^Heinz Maier-Leibnitz Zentrum (MLZ), Technical University Munich, Lichtenbergstr. 1, 85748 Garching, Germany

*Tatsuya Joutsuka, *Masaki Unno

Email: tatsuya.joutsuka.joe@vc.ibaraki.ac.jp, masaki.unno.19@vc.ibaraki.ac.jp

**^‡^** These authors contributed equally.

A list of the material included: Supporting text, Figures S1 to S7, Table S1, SI References


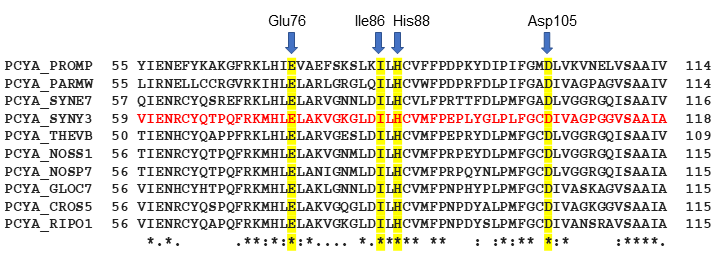


Figure S1. Conserved amino acid residues of the PcyA family from several species. Red letters indicate amino acid residues of PcyA from *Synechocystis* sp. PCC 6803. Yellow-highlighted letters indicate key amino acid residues of interest in the present study, which are well conserved across species.


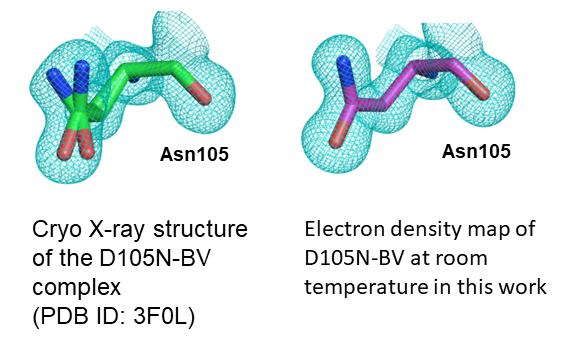


Figure S2. Electron density maps and structure models for Asn105 in the D105N-BV complex. Left: Cryo X-ray structure (PDB ID: 3F0L), in which Asn105 can be modeled as two conformations. Right: Joint-refined structure of Asn105 in a single conformation.


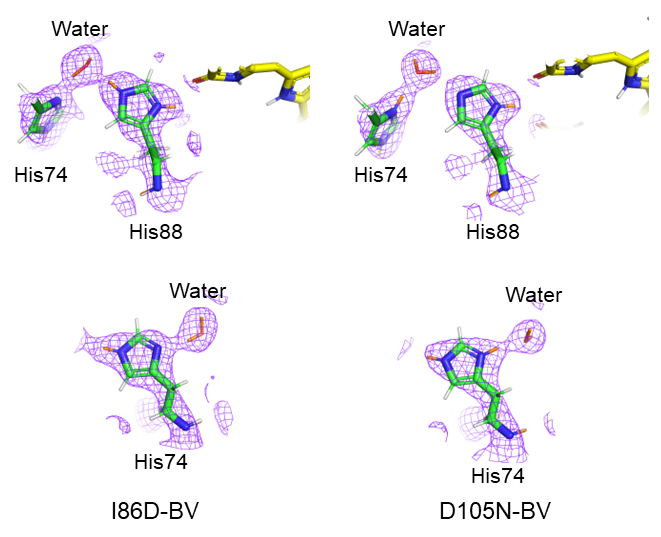


Figure S3. 2*F*_o_ – *F*_c_ neutron scattering length maps and structure models for His88, His74, and the intervening water molecule in the I86D-BV complex (left) and D105N-BV complex (right). The maps are contoured at 1.8σ level. Carbon, oxygen, and nitrogen atoms are colored green, red, and blue, respectively in protein molecules. Carbon atoms in BV are colored yellow. Hydrogen and deutrerium atoms are colored white and orange, respectively.


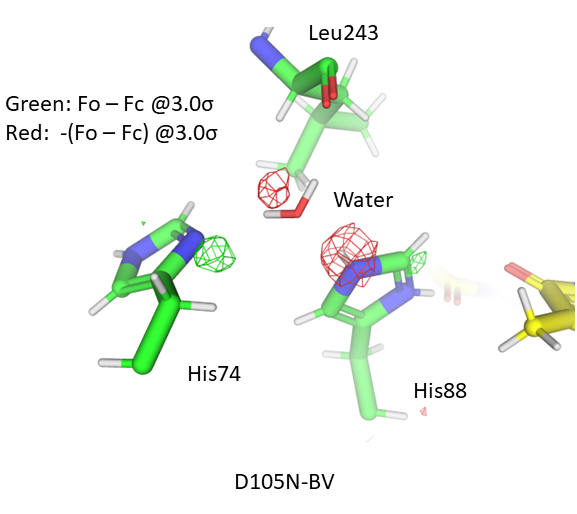


Figure S4. Protonation states of His74 and His88 in the D105N-BV complex. Green and red cages represent +(*F*_o_–*F*_c_) and –(*F*_o_–*F*_c_) neutron-scattering density maps at 3.0σ levels, when His88Nε is modeled as a protonated state.

Computational Details of Absorption Spectra via QM/MM Method

As the experimental absorption spectra were measured in solution, we used the water cap that spherically surrounds the entire protein, with a radius of 40.0 Å, from BV, as shown in Figure S4A. Six (WT), nine (I86D), and seven (D105) Na^+^ ions were included for neutralization of the system by using the Antechamber program (1). We note that a water molecule next to His88 and His74 was not modeled as H_3_O^+^, but as H_2_O, because the recent computational studies suggest the preference of H_2_O (2,3). It is further noted that after geometry optimization, H_2_O did not transform to H_3_O^+^ by protonation from the neighboring His residues.

Our own N-layered integrated molecular orbital and molecular mechanics (ONIOM) (4,5) method was employed in this study for QM/MM calculations. All ONIOM calculations were performed using Gaussian 16 Revision C.01 (6). Geometry optimization was conducted using the density functional method with the M06-2X (7) exchange-correlation functional and 6-31G(d) basis set (see below for the functional and basis set dependence) for the QM region, whereas the AMBER force field (8) implemented in Gaussian 16 (6) was employed for the MM region. An electrostatic embedding scheme was applied to all calculations (4). The QM region of the WT-BV complex shown in Figure S4B includes BV, Glu76, His88, His74, Asp105, Asn219, Thr222, Leu243, and three water molecules. Only the side chains of all the amino acids (except BV and water molecules) were included in the QM region. For instance, 4-methylimidazole in His residues and acetic acid in Glu and Asp residues were assigned as QM atoms. The other atoms in the complex were treated at the MM level. The I86D-BV in Figure S4C includes an additional Asp86 and a water molecule nearby, but it excludes one water molecule close to Asn219 and Thr222 for the QM region, as in the crystal structure. D105N-BV (Figure S4D) includes acetamide (from the asparagine residue) and three water molecules near Asn219 and Thr222 in the QM region. The number of atoms in the QM region (excluding the link atoms) was 144 for WT-BV, 151 for I86D-BV, and 145 for D105N-BV. In D105N-BV, the protonation of asparagine was kept fixed.

The coordinates of the solvent molecules were first optimized to eliminate distortion in the initial geometry, with the MM region of PcyA fixed by QM/MM calculations. Using the optimized solvent geometry, we prepared the initial geometry for each protonation state, as shown in Figure 7 in the main text. Only the coordinates of the QM region and atoms covalently bonded to the QM region were relaxed. In contrast, the other atoms in the MM region were fixed during geometry optimization. The initial structures changed for the protonation states, as shown in Figure 7 in the main text.

After the geometry optimization of each protonation state, time-dependent density functional theory (TD-DFT) calculations (9) were performed to calculate the absorption spectra, with 40 excited states solved using the optimized geometry discussed above. The absorption line shapes were obtained using GaussView (10) with a line width of 0.33 eV. The computational conditions of the TD-DFT calculations, such as the QM region and DFT functional, remained the same as those for geometry optimization.

In addition to the M06-2X (7) functional, geometry optimization was conducted with the CAM-B3LYP (11) B3LYP (12) , PBE0 (13) , and LC-ωHPBE (14) exchange-correlation functionals, and absorption spectra were calculated using TD-DFT (9) and the same functional. Figure S5 shows the functional dependence of the absorption spectra of the B protonation state of the WT-BV complex. Among the employed functionals, M06-2X reproduces the Q band at approximately 700 nm. CAM-B3LYP also performs relatively well in the Q band, whereas LC-ωHPBE underestimates the peak position overall. B3LYP and PBE0 exhibited no peaks at approximately 400 nm in the Soret band. Considering its agreement with the M06-2X functional, we employed the M06-2X functional in this study.

Furthermore, using the M06-2X functional, the peak centers of WT-BV are between 600 and 700 nm, which is slightly smaller than the experimental values (approximately 590, 660, and 730 nm) (15,16). This may imply that all spectra should be shifted by +75 nm, approximately. Thus, we employed the M06-2X functional and shifted the computed spectra by +75 nm for a clearer comparison with the experimental results in the following calculations.

In addition, Figure S6 shows the computed absorption spectra with the M06-2X/6-31G(d) and M06-2X/Def2SVP levels (17). The difference is minor compared to the functional difference. Therefore, we employed M06-2X/6-31G(d) in our study.

Figure S7 shows the differences between the electronic and mechanical embeddings in the QM/MM calculations. Geometry optimizations and subsequent single-point calculations of the absorption spectrum were performed using the same embedding scheme. Mechanical embedding yields a small tail in the absorption spectrum, which differs from that in the experiment. Thus, we only employed electronic embedding in this study.

Table S1 summarizes the details of the computed absorption spectra of the PcyA-BV complex.


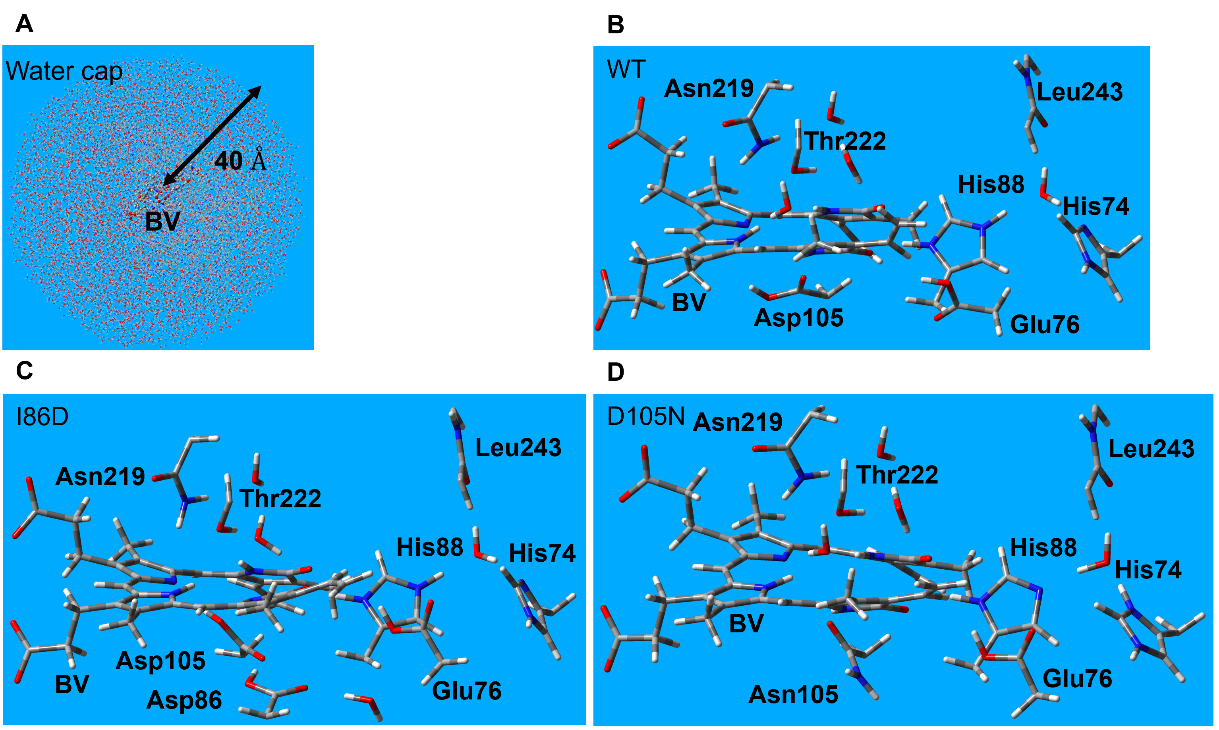


Figure S5. Simulation snapshot of (a) the entire protein and solvent, as well as QM region of the (b) wild type (WT), (c) I86D, and (d) D105N employed in the QM/MM calculation. QM and MM regions are represented as ball-and-stick models and lines, respectively. These snapshots were created using GaussView (10).


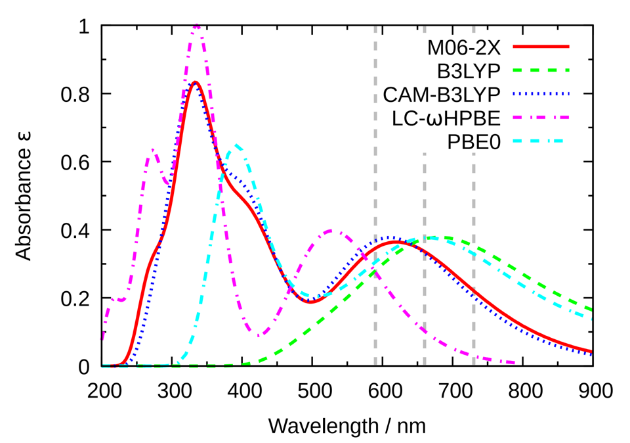


Figure S6. Functional dependence of the computed absorption spectra for the B protonation state of the WT-BV complex. The gray dashed lines denote the reference peak positions from experiments at 590, 660, and 730 nm.


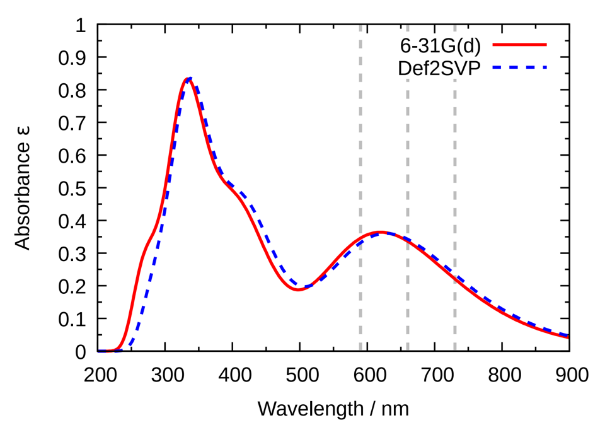


Figure S7. Basis set dependence of the computed absorption spectra using the M06-2X functional for the B protonation state of WT-BV.


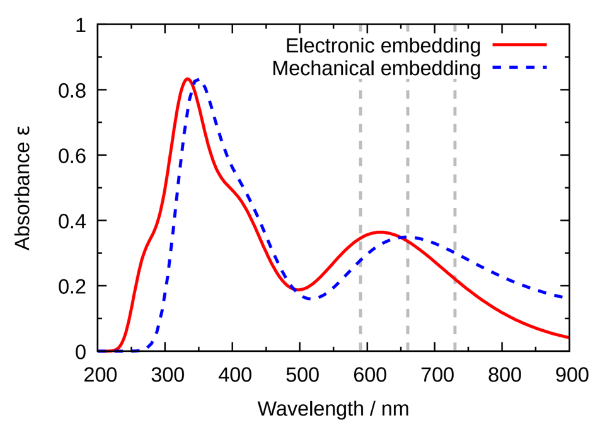


Figure S8. Effect of electronic embedding, compared to mechanical embedding, in the computed absorption spectra using the M06-2X functional for the B protonation state of WT-BV.

Table S1. Wavelength ($\boldsymbol{\lambda}$, nm), the corresponding oscillator strengths ($\boldsymbol{f}$), and description of the dominant configuration in the computed absorption spectra.

| **PcyA** | $\boldsymbol{\lambda}$ | $\boldsymbol{f}$ | **%** | **character** |
| --- | --- | --- | --- | --- |
| WT (B) | 640 | 0.3399 | 67 | HOMO → LUMO |
|  |  |  | 22 | HOMO-2 → LUMO |
|  | 422 | 0.3046 | 63 | HOMO → LUMO+1 |
|  |  |  | 24 | HOMO-2 → LUMO+1 |
|  | 335 | 0.7746 | 86 | HOMO-9 → LUMO |
|  |  |  | 14 | HOMO-13 → LUMO+1 |
| WT (C) | 629 | 0.2597 | 73 | HOMO → LUMO |
|  |  |  | 21 | HOMO-3 → LUMO |
|  | 419 | 0.2288 | 79 | HOMO → LUMO+1 |
|  |  |  | 13 | HOMO-3 → LUMO+1 |
|  | 332 | 0.6364 | 78 | HOMO-8 → LUMO |
|  |  |  | 8 | HOMO-7 → LUMO |
| D105N (C with deproton. Glu76) | 552 | 0.3673 | 96 | HOMO → LUMO |
|  |  |  | 2 | HOMO-1 → LUMO+1 |
|  | 390 | 0.3705 | 89 | HOMO → LUMO+1 |
|  |  |  | 8 | HOMO-1 → LUMO |
|  | 328 | 0.9031 | 37 | HOMO-1 → LUMO |
|  |  |  | 37 | HOMO-4 → LUMO |
| D105N (C) | 613 | 0.3892 | 95 | HOMO → LUMO |
|  | 415 | 0.3845 | 83 | HOMO → LUMO+2 |
|  |  |  | 8 | HOMO-2 → LUMO |
|  | 335 | 0.7442 | 37 | HOMO-3 → LUMO |
|  |  |  | 26 | HOMO-5 → LUMO |

**SI References**

1. Wang, J., Wang, W., Kollman, P. A., and Case, D. A. (2006) Automatic atom type and bond type perception in molecular mechanical calculations. *Journal of Molecular Graphics and Modelling* **25**, 247-260

2. Iijima, E., Gleeson, M. P., Unno, M., and Mori, S. (2018) QM/MM Investigation for Protonation States in a Bilin Reductase PcyA-Biliverdin IXɑ Complex. *ChemPhysChem* **19**, 1809-1813

3. Ikeda, T., Saito, K., Hasegawa, R., and Ishikita, H. (2017) The Existence of an Isolated Hydronium Ion in the Interior of Proteins. *Angew Chem Int Ed Engl* **56**, 9151-9154

4. Vreven, T., Byun, K. S., Komáromi, I., Dapprich, S., Montgomery, J. A., Morokuma, K., and Frisch, M. J. (2006) Combining Quantum Mechanics Methods with Molecular Mechanics Methods in ONIOM. *Journal of Chemical Theory and Computation* **2**, 815-826

5. Chung, L. W., Sameera, W. M. C., Ramozzi, R., Page, A. J., Hatanaka, M., Petrova, G. P., Harris, T. V., Li, X., Ke, Z., Liu, F., Li, H.-B., Ding, L., and Morokuma, K. (2015) The ONIOM Method and Its Applications. *Chemical Reviews* **115**, 5678-5796

6. Frisch, M. J., Trucks, G. W., Schlegel, H. B., Scuseria, G. E., Robb, M. A., Cheeseman, J. R., Scalmani, G., Barone, V., Petersson, G. A., Nakatsuji, H., Li, X., Caricato, M., Marenich, A. V., Bloino, J., Janesko, B. G., Gomperts, R., Mennucci, B., Hratchian, H. P., Ortiz, J. V., Izmaylov, A. F., Sonnenberg, J. L., Williams, Ding, F., Lipparini, F., Egidi, F., Goings, J., Peng, B., Petrone, A., Henderson, T., Ranasinghe, D., Zakrzewski, V. G., Gao, J., Rega, N., Zheng, G., Liang, W., Hada, M., Ehara, M., Toyota, K., Fukuda, R., Hasegawa, J., Ishida, M., Nakajima, T., Honda, Y., Kitao, O., Nakai, H., Vreven, T., Throssell, K., Montgomery Jr., J. A., Peralta, J. E., Ogliaro, F., Bearpark, M. J., Heyd, J. J., Brothers, E. N., Kudin, K. N., Staroverov, V. N., Keith, T. A., Kobayashi, R., Normand, J., Raghavachari, K., Rendell, A. P., Burant, J. C., Iyengar, S. S., Tomasi, J., Cossi, M., Millam, J. M., Klene, M., Adamo, C., Cammi, R., Ochterski, J. W., Martin, R. L., Morokuma, K., Farkas, O., Foresman, J. B., and Fox, D. J. (2016) Gaussian 16 Rev. C.01. Wallingford, CT

7. Zhao, Y., and Truhlar, D. G. (2008) The M06 suite of density functionals for main group thermochemistry, thermochemical kinetics, noncovalent interactions, excited states, and transition elements: two new functionals and systematic testing of four M06-class functionals and 12 other functionals. *Theoretical Chemistry Accounts* **120**, 215-241

8. Cornell, W. D., Cieplak, P., Bayly, C. I., Gould, I. R., Merz, K. M., Ferguson, D. M., Spellmeyer, D. C., Fox, T., Caldwell, J. W., and Kollman, P. A. (1995) A Second Generation Force Field for the Simulation of Proteins, Nucleic Acids, and Organic Molecules. *Journal of the American Chemical Society* **117**, 5179-5197

9. Adamo, C., and Jacquemin, D. (2013) The calculations of excited-state properties with Time-Dependent Density Functional Theory. *Chemical Society Reviews* **42**, 845-856

10. Roy, D., Todd, A. K., and John, M. M. (2016) GaussView Version 6.

11. Yanai, T., Tew, D. P., and Handy, N. C. (2004) A new hybrid exchange–correlation functional using the Coulomb-attenuating method (CAM-B3LYP). *Chemical Physics Letters* **393**, 51-57

12. Becke, A. D. (1993) Density‐functional thermochemistry. III. The role of exact exchange. *The Journal of Chemical Physics* **98**, 5648-5652

13. Adamo, C., and Barone, V. (1999) Toward reliable density functional methods without adjustable parameters: The PBE0 model. *The Journal of Chemical Physics* **110**, 6158-6170

14. Henderson, T. M., Izmaylov, A. F., Scalmani, G., and Scuseria, G. E. (2009) Can short-range hybrids describe long-range-dependent properties? *The Journal of Chemical Physics* **131**, 044108

15. Tu, S. L., Gunn, A., Toney, M. D., Britt, R. D., and Lagarias, J. C. (2004) Biliverdin reduction by cyanobacterial phycocyanobilin:ferredoxin oxidoreductase (PcyA) proceeds via linear tetrapyrrole radical intermediates. *J Am Chem Soc* **126**, 8682-8693

16. Unno, M., Ishikawa-Suto, K., Kusaka, K., Tamada, T., Hagiwara, Y., Sugishima, M., Wada, K., Yamada, T., Tomoyori, K., Hosoya, T., Tanaka, I., Niimura, N., Kuroki, R., Inaka, K., Ishihara, M., and Fukuyama, K. (2015) Insights into the Proton Transfer Mechanism of a Bilin Reductase PcyA Following Neutron Crystallography. *J Am Chem Soc* **137**, 5452-5460

17. Schäfer, A. H., H.; Ahlrichs, R. . (1992) Fully optimized contracted Gaussian basis sets for atoms Li to Kr. *J. Chem. Phys.* **97**, 2571-2577
